# Nanofactory for metabolic and chemodynamic therapy: pro-tumor lactate trapping and anti-tumor ROS transition

**DOI:** 10.1186/s12951-021-01169-9

**Published:** 2021-12-18

**Authors:** Ruiqing He, Jie Zang, Yuge Zhao, Ying Liu, Shuangrong Ruan, Xiao Zheng, Gaowei Chong, Dailin Xu, Yan Yang, Yushan Yang, Tingting Zhang, Jingjing Gu, Haiqing Dong, Yongyong Li

**Affiliations:** 1 Shanghai Skin Disease Hospital, The Institute for Biomedical Engineering & Nano Science, School of Medicine, Tongji University, 200092 Shanghai, China; 2 Shanghai East hospital, School of Medicine, Tongji University, 200092 Shanghai, China

**Keywords:** Lactate, Immunosuppressive tumor microenvironment, Immunogenic cell death, Chemodynamic therapy

## Abstract

Lactate plays a critical role in tumorigenesis, invasion and metastasis. Exhausting lactate in tumors holds great promise for the reversal of the immunosuppressive tumor microenvironment (TME). Herein, we report on a “lactate treatment plant” (i.e., nanofactory) that can dynamically trap pro-tumor lactate and in situ transformation into anti-tumor cytotoxic reactive oxygen species (ROS) for a synergistic chemodynamic and metabolic therapy. To this end, lactate oxidase (LOX) was nano-packaged by cationic polyethyleneimine (PEI), assisted by a necessary amount of copper ions (PLNP^Cu^). As a reservoir of LOX, the tailored system can actively trap lactate through the cationic PEI component to promote lactate degradation by two-fold efficiency. More importantly, the byproducts of lactate degradation, hydrogen peroxide (H_2_O_2_), can be transformed into anti-tumor ROS catalyzing by copper ions, mediating an immunogenic cell death (ICD). With the remission of immunosuppressive TME, ICD process effectively initiated the positive immune response in 4T1 tumor model (88% tumor inhibition). This work provides a novel strategy that rationally integrates metabolic therapy and chemodynamic therapy (CDT) for combating tumors.

**Supplementary Information:**

The online version contains supplementary material available at 10.1186/s12951-021-01169-9.

## Background

The tumor microenvironment (TME) is critically important during the initiation and progression of carcinogenesis [1]. Lactate, has long been considered as the waste product of tumor aerobic glycolysis (termed as the “Warburg effect”), is now emerged as an important regulator of tumor development [2]. Increasing evidence has demonstrated that lactate acted as accomplices to assist tumor immune escape and promote tumor progression, especially through suppressing the function of multiple immune cells, such as macrophages, dendritic cells (DCs), effector T cells [3–5]. Therefore, to against cancer, several agents (glycolysis inhibitors (Dichloroacetate, DCA), MCT-inhibiting siRNA (siMCT-4) et al.) have been delivered via nanocarriers to inhibit lactate production and transportation [6, 7]. Lactate oxidase (LOX), catalyzing the oxidation of lactate to pyruvate, has also been harnessed for lactate degradation and TME regulation [8–10].

The LOX delivery based on nanocarrier represents a promising approach, as it addresses the bio-stability and side effect challenges [8]. Unfortunately, it is still facing the dilemma that the inefficiency of lactate depletion in complex TME and tumor inhibition. Most current works focused on the decomposing lactate with LOX, combined with biozym (glucose oxidase, GOx) or photodynamic therapy to enhance the anti-tumor effect, in which the introduction of additional therapeutic cargo complicated the design of nanomedicine [11, 12]. From a different perspective, we propose a strategy of “turn waste into wealth”, developed to not only exhaust the lactate but also transform the hydrogen peroxide (H_2_O_2_), the byproduct of lactate degradation, into cytotoxic drugs via a catalytic medicine approach.

The decomposition of lactate with LOX produces H_2_O_2_, [13] which can be harnessed to realize Fenton reaction with Fenton agents (e.g., Cu^2+^, Fe^3+^/Fe^2+^) to produce more cytotoxic hydroxyl radicals (·OH) for tumor killing, namely chemodynamic therapy (CDT) [14]. Meanwhile, excess reactive oxygen species (ROS) induce immunogenic cancer cell death (ICD), and promote the secretion of various pro-inflammatory cytokines from immune cells, (e.g., interferon-γ (IFN-γ), interleukin-6 (IL-6), tumor necrosis factor (TNF)), thereby enhancing the inflammatory response in tumor sites for further immune activation [15]. Since Fe^2+^-catalyzed Fenton reaction efficiency was restrained by strongly acidic conditions (pH 2−4). Cu^2+^, possessing a higher reaction rate of ∼160-fold of Fe^2+^ in the weakly acidic and neutral media, could be a better choice [14, 16–18].

**Scheme 1:**
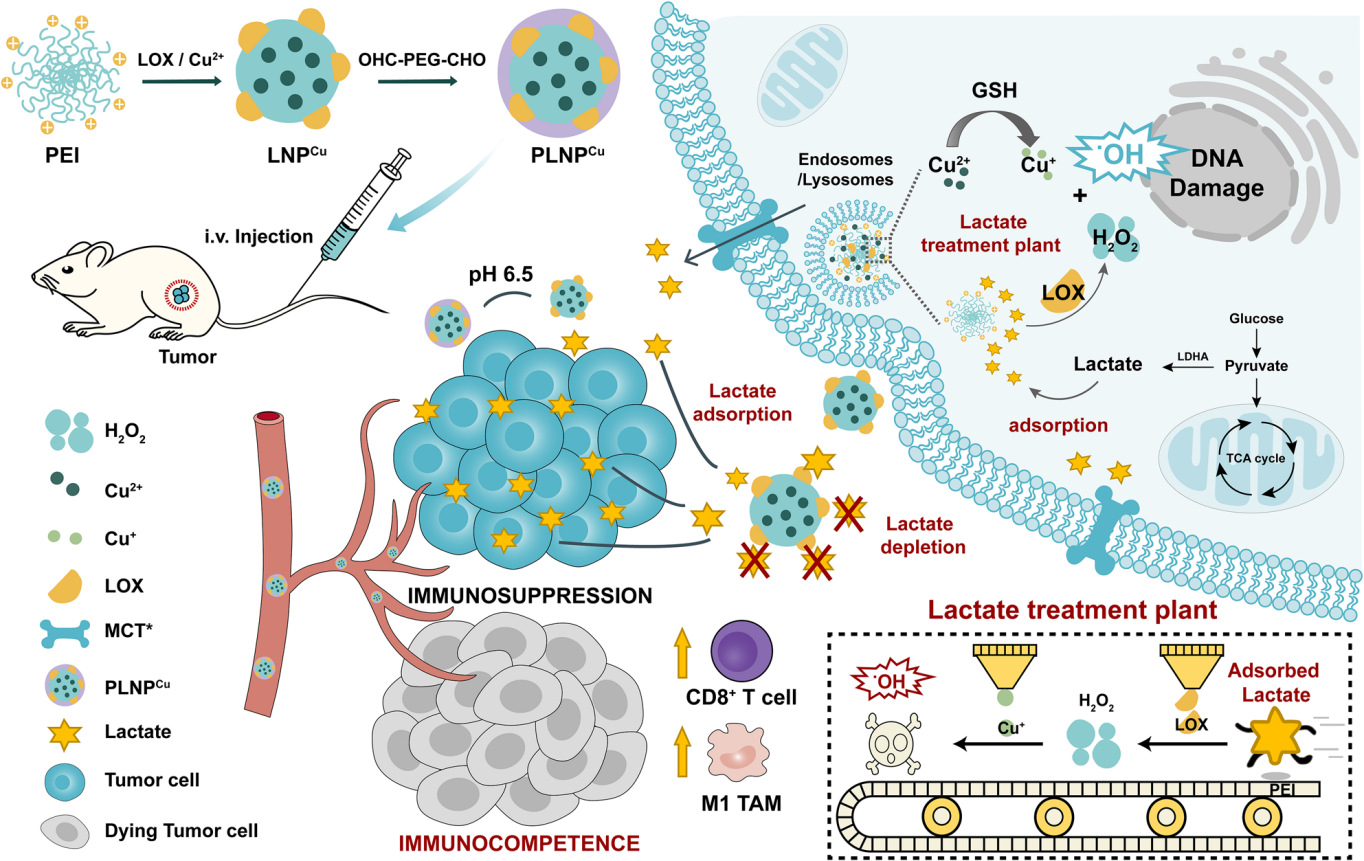
Schematic illustration of the lactate exhaustion process of PLNP^Cu^ nanosystem through extracellular lactate active-adsorption and establishment of the intracellular lactate treatment plant

Accordingly, herein, we developed the “lactate treatment plant” (i.e., nanofactory) that can dynamically trap pro-tumor lactate and in situ transformation into anti-tumor cytotoxic ROS for a synergistic chemodynamic and metabolic therapy. As shown in Scheme 1, cationic polyethyleneimine (PEI), possessing robust acid trapping ability, was chosen as the carrier for loading LOX and Cu^2+^ through electrostatic interaction and coordination, named as LNP^Cu^. The pH-responsive detachable polyethylene glycol (OHC-PEG-CHO) shell endows LNP^Cu^ with stability and biocompatibility (PLNP^Cu^). Upon acid-triggered shell shedding, lactate gathered surrounding the “nanofactory” through the “active trapping” of the exposed primary amine on PEI. Then the LOX contained in “nanofactory” facilitated the lactate catalysis to generate H_2_O_2_ which was continuously transformed into toxic ·OH through the Fenton-like reaction. In this fashion, the lactate depletion by the cascade catalysis process reversed lactate-induced suppression of immune cells function in TME, and more importantly, its product of ROS subsequently induced ICD and anti-tumor immunity. Therapeutically, the “lactate treatment plant” significantly inhibited the growth of 4T1 breast cancer.

## Results and discussion

### Synthesis and characterization of PLNP^Cu^

The LNP^Cu^ can quickly assemble by mixing polyethyleneimine (PEI), copper ions (Cu^2+^) and lactate oxidase (LOX) under gentle stirring at room temperature (see supporting information for the detailed description of the preparation). The driving force for the assembly was mainly electrostatic interaction and coordination among Cu^2+^, PEI and LOX. The obtained LNP^Cu^ have the hydrodynamic size of 115 ± 5.0 nm with a narrow polydispersity index (PDI) of 0.14 by dynamic light scattering (DLS) (Additional file 1: Fig. S1d).

**Fig. 1:**
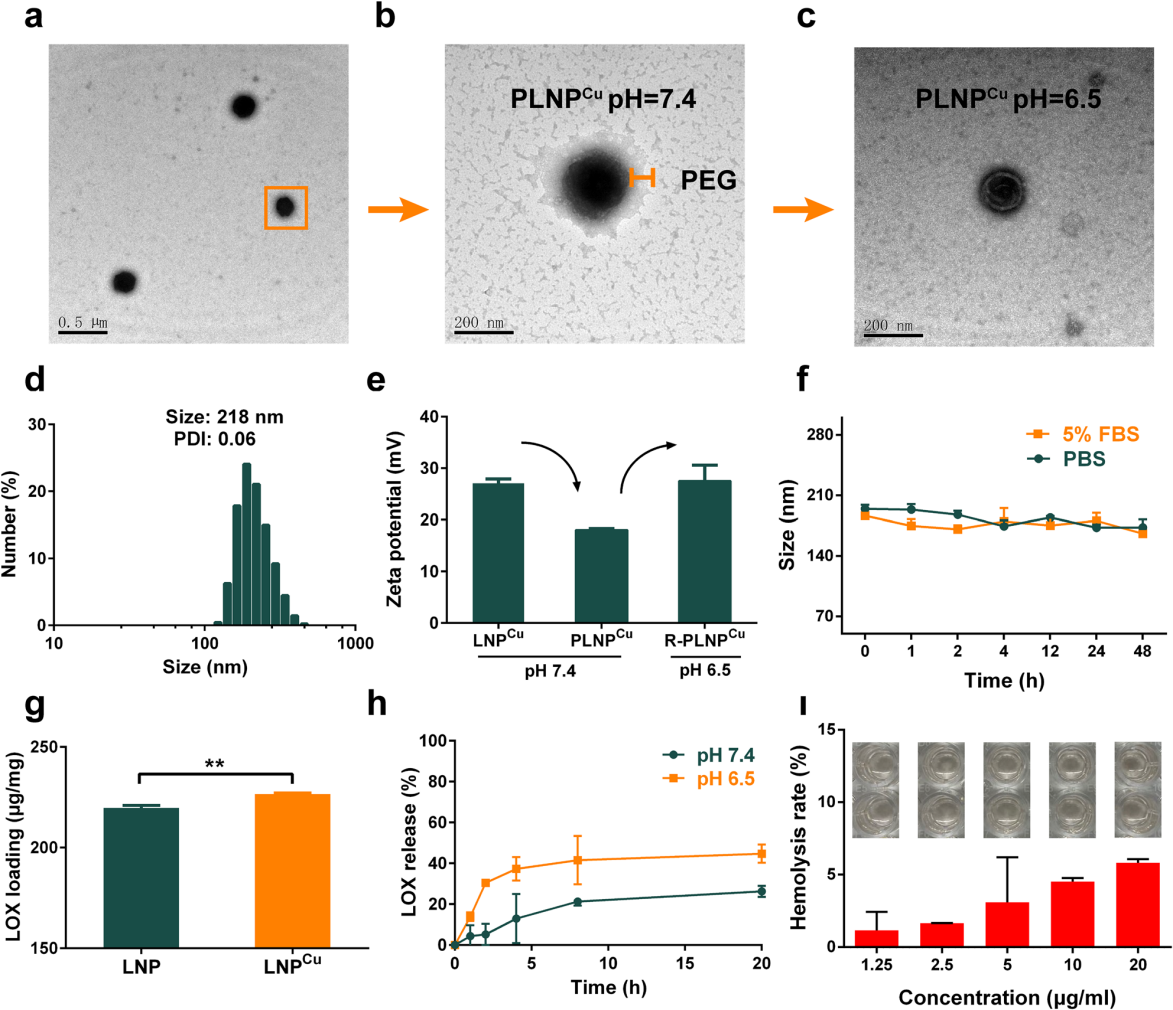
Characterization of PLNP^Cu^. **a**, ** b** TEM images of PLNP^Cu^ at pH 7.4. **c** TEM images of PLNP^Cu^ at pH 6.5. **d** DLS of PLNP^Cu^ at pH 7.4. **e** The pH-triggered charge rebound behavior of PLNP^Cu^ (n = 3). **f** Maintenance of size stability of PLNP^Cu^ in PBS and PBS with 5% FBS (at pH 7.4) (n = 3). **g** LOX loading capacities on LNP and LNP^Cu^ respectively (n = 3). **h** Cumulative release of LOX from PLNP^Cu^ in PBS at pH 7.4 and pH 6.5 (n = 3). **i** Hemolysis rate of PLNP^Cu^ at different PEI concentrations (n = 3). Results were expressed as mean ± SD. The significant difference was calculated via two-tailed t-test analysis (**g**). (NS represented not significant, *p < 0.05, **p < 0.01, ***p < 0.001)

To improve the circulation stability and biocompatibility, the surface of the LNP^Cu^ was shielded with polyethylene glycol derivates with the double end group of aldehyde (OHC-PEG-CHO) through pH-responsive Schiff base (named as PLNP^Cu^). As shown in the ^1^H NMR spectra, the disappearance (at pH 7.4) and reappearance (at pH 6.5) of the hydrogen peak for aldehyde groups in the dotted area suggested that the acid-responsive labiality of Schiff bases formed by PEI with aldehyde groups of OHC-PEG-CHO (Additional file 1: Fig. S4). PLNP^Cu^ showed well-dispersed uniform spherical structures (Fig. 1a) with a visible PEG shell at pH 7.4 by transmission electron microscopy (TEM) (Fig. 1b). The hydrodynamic size of PLNP^Cu^ was 218 ± 3.0 nm (PDI 0.06), which was consistent well with the TEM results (Fig. 1d). Notably, PLNP^Cu^ exhibited the compression of zeta potential comparing with LNP^Cu^ (27.07 mV vs. 18.07 mV) (Fig. 1e). However, the rebound of zeta potential from 18.07 mV to 27.50 mV (Fig. 1e) occurred at the weakly acidic condition (at pH 6.5, the pH of most solid tumor environment) [19], indicating PEG shell could be shed from the surface of PLNP^Cu^ to exposure of the positive charged amino. More intuitively, the shellless PLNP^Cu^ upon incubated in H_2_O at pH 6.5 could be viewed in TEM images, as was shown in Fig. 1c and Additional file 1: Fig. S1a.

Further, the size of PLNP^Cu^ was stable in neutral PBS solution within 48-hour monitoring (Fig. 1f) accompanied by a low release of LOX (26.25% ± 2.676) (Fig. 1 h), the content of LOX was quantified with the Bradford Protein Assay Kit according to the standard curve (Additional file 1: Fig. S3), while the moderate LOX release behavior was found at pH 6.5 (44.74% ± 4.456) (Fig. 1h). The morphology of the PLNP^Cu^ at pH 5.5 was also explored (Additional file 1: Fig. S1b), which suggested the potential for effective function exertion in the intracellular milieu. Apparently, the introduction of copper ion (with entrapment efficiency of ∼81.58% determined by inductively coupled plasma mass spectrometry (ICP-MS)) may facilitate LOX loading in LNP^Cu^ compared with the LNP with a smaller size (Additional file 1: Fig. S1c) which could be attributed to the coordination of metal ions (LOX %: 226.6 ± 0.518 vs. 219.7 ± 1.237 µg mg^−1^) (Fig. 1g). In addition, hemolytic toxicity tests showed the biosafety of PLNP^Cu^ through intravenous administration (Fig. 1i).

### Evaluation of PLNP^Cu^ as lactate treatment plant in vitro

**Scheme 2:**
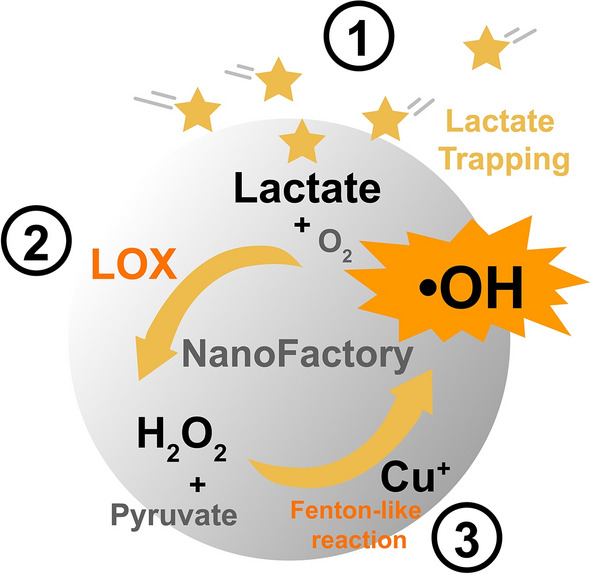
Schematic illustration of the PLNP^Cu^ nanofactory closed-loop for lactate consumption and conversion

PLNP^Cu^, referred to as extra/intracellular lactate treatment plant, for lactate consumption and tumor cell inhibition were investigated. As shown in Scheme 2, the lactate treatment plant elicited a chain of the response of processing lactate to antipersonnel reactive oxygen species (ROS) capitalized on the tumor cell physiology and tumor microenvironments (TME), such as acidic microenvironment and the relatively high GSH/GSSG ratios [20, 21]. Briefly, the entire process can be categorized into three stages. First, the local high lactate concentration is formed by PEI trapping; second, lactate reacts with oxygen to produce hydrogen peroxide (H_2_O_2_) under the catalysis of LOX; finally, the Fenton-like reaction between copper ions and enzymatic product (H_2_O_2_) from the second step produces a large number of hydroxyl radicals (·OH). The verification for the above three stages was investigated as follows.

**Fig. 2:**
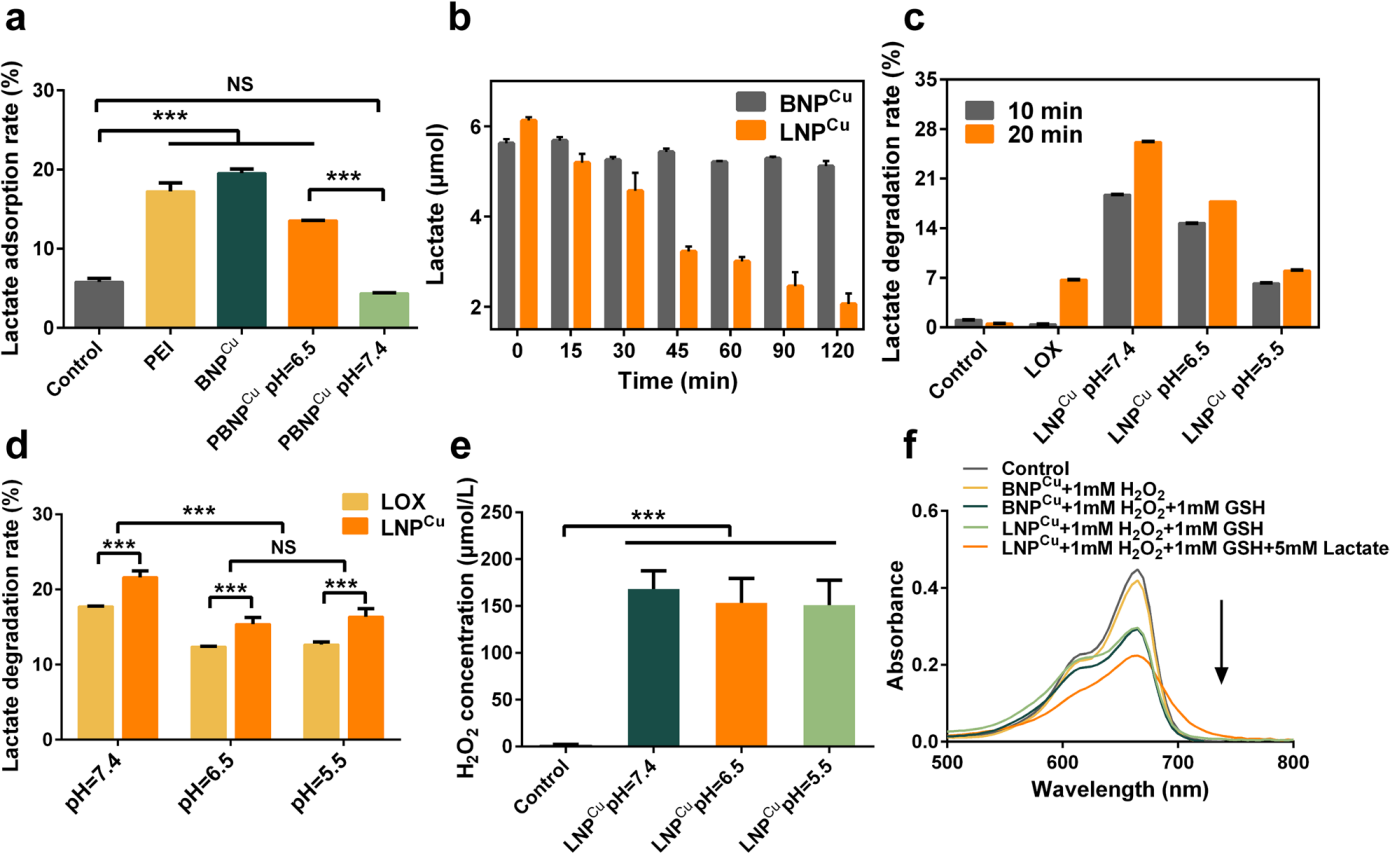
Functions verification of lactate treatment plant PLNP^Cu^ in vitro. **a** Lactate adsorption rate of PEI in nanoparticles (n = 3). **b** Lactate depletion effect of LNP^Cu^ overtime at pH 7.4 (n = 3). **c** Lactate degradation ratio of LNP^Cu^ at the shorter incubation time in different acidic solutions (n = 3). **d** Lactate degradation rate of LNP^Cu^ compared with LOX after sufficiently enzymatic hydrolysis (n = 3). **e** The generation of enzymatic product H_2_O_2_ (n = 3). **f** The degradation process of MB caused by ·OH generation from the Fenton-like reaction of nanoparticles under different conditions. Results were expressed as mean ± SD. The significant difference was calculated via one-way ANOVA analysis (**a**, **d**, **e**). (NS represented not significant, *p < 0.05, **p < 0.01, ***p < 0.001)

Inspired by the cationic polymer of PEI with a large number of primary amino groups, we speculated that these positive charged groups could electrostatically interact with lactate and attract to the PLNP^Cu^ nanosystem. We thus carefully studied the adsorption behavior of PLNP^Cu^. Considering the LOX may influence the determination of lactate absorption of PLNP^Cu^, bovine serum albumin (BSA) was used to replace LOX, regarding their high similarity in structure and properties (isoelectric point, pI 4.7 vs. 4.6, molecular weight ≈ 66 kDa vs. 80 kDa) except for catalysis for lactate, and was fabricated according to the synthetic procedure of LNP^Cu^ and PLNP^Cu^ (named as BNP^Cu^ and PBNP^Cu^, respectively). Both zeta potential and hydrodynamic size of BNP^Cu^ and PBNP^Cu^ were consistent with PLNP^Cu^ (Additional file 1: Fig. S2b). Subsequently, the PEI, BNP^Cu^, and PBNP^Cu^ at different pH with an equimolar amount of PEI were mixed with NaL respectively and shaken overnight at 37 ℃. Followed by fully dialyzed for 4 h and the dialysate of different groups was collected for lactate analysis. The high lactate adsorption rate of BNP^Cu^ (19.54% ± 0.559) and PBNP^Cu^ (pH = 6.5) (13.56% ± 0.0284) were consistent with that of PEI solution (17.24% ± 1.07), while the PBNP^Cu^ (4.369% ± 0.0583) and control groups (5.804% ± 0.429) at pH 7.4 showed much lower levels of lactate adsorption compared to above three groups (Fig. 2a). The above results verified that the exposure of PEI on the PLNP^Cu^ nanosystem played an important role in lactate recruitment, and the detachable PEG shell contributed to the shielding effect in the neutral milieu.

To evaluate the lactate consumption-ability of the nanoparticles, LNP^Cu^ and BNP^Cu^ were added into NaL solution respectively. The concentration of lactate in the mixed solution containing LNP^Cu^ decreased with time compared to BNP^Cu^ (Fig. 2b), which demonstrated the maintenance of the catalytic activity of LOX. We further studied the degradation of lactate over a short time frame (10 or 20 min) at different pH. Surprisingly, after being incubated for 20 min, the lactate degradation rate of LNP^Cu^ was almost four-fold higher than the free LOX group at pH 7.4 (26.16% ± 0.102 vs. 6.727% ± 0.0298), even reaching the ~43 times level in 10 min of reaction (18.71% vs. 0.429%). And LNP^Cu^ exhibited an excellent catalytic activity even at pH 5.5 (7.977% ± 0.122) (Fig. 2c). Immediately after, the degradation rate of lactate following 2 h exposure to LNP^Cu^ was studied. The result showed that the catalytic activity of LNP^Cu^ at different pH values (pH 7.4, 6.5, and 5.5) was significantly higher than the respective values of free LOX groups (Fig. 2d). It was speculated that the discrepancy was likely because of the adsorption effect of PEI in favor of shortening the “lactate-hunting” for LOX, and local high concentration of lactate was more favorable for the performance of enzymatic hydrolysis reaction. The above results indicated that the lactate adsorption effect of PEI contributed to the enhance of lactate degradation at different pH.

Based on the above exciting results, the third process in the lactate treatment plant that ·OH production by reacting H_2_O_2_ with Cu^+^ was explored. The concentration of H_2_O_2_ was quantified via the Hydrogen Peroxide Assay Kit according to the standard curve (Additional file 1: Fig. S5). The LOX solution was mixed with NaL solution and thoroughly reacted for 1 h at 37 ℃ and then the H_2_O_2_ concentration of the supernatant was detected. As shown in Additional file 1: Fig. S6, H_2_O_2_ was barely detected without NaL, which has excluded the effect of residual H_2_O_2_ background. And the generation of H_2_O_2_ increased along with increasing NaL concentration. This directly demonstrated the LOX catalyzed reaction of lactate + O_2_ — pyruvate + H_2_O_2_. After that, the generation of H_2_O_2_ produced by LNP^Cu^ at different pH conditions (pH 7.4, 6.5 and 5.5) compared with free LOX solution was recorded. The H_2_O_2_ concentration was comparable in both groups which demonstrated carrier and pH change did not show interference in this process (Fig. 2e and Additional file 1: Fig. S7). The methylene blue (MB) degradation was used to validate the Fenton-like reaction occurrence. We simulated the process of intracellular high levels of GSH (5–10 mM) induced the conversion of cupric ions (Cu^2+^) to cuprous form (Cu^+^) [20]. Briefly, GSH was added into the supernatant of BNP^Cu^ and LNP^Cu^ solution respectively for Cu^+^ generation, then the H_2_O_2_ and NaL solution were added in turn. The appropriate feeding amount of H_2_O_2_, GSH and NaL was identified from references [22, 23]. The reduction in absorbance of MB showed that additional lactate evoked a huge elevation of toxic ·OH in the orange group (with lactate addition) compared to light green groups (without lactate addition) (Fig. 2f). It was proven that the Cu^+^ resulted from GSH reduction can react with H_2_O_2_ to produce ·OH, since the ·OH-induced MB indicator degradation caused the change in absorbance [22]. All the results supported a well-functioning lactate treatment plant in vitro.

### Intracellular behaviors of PLNP^Cu^

**Fig. 3:**
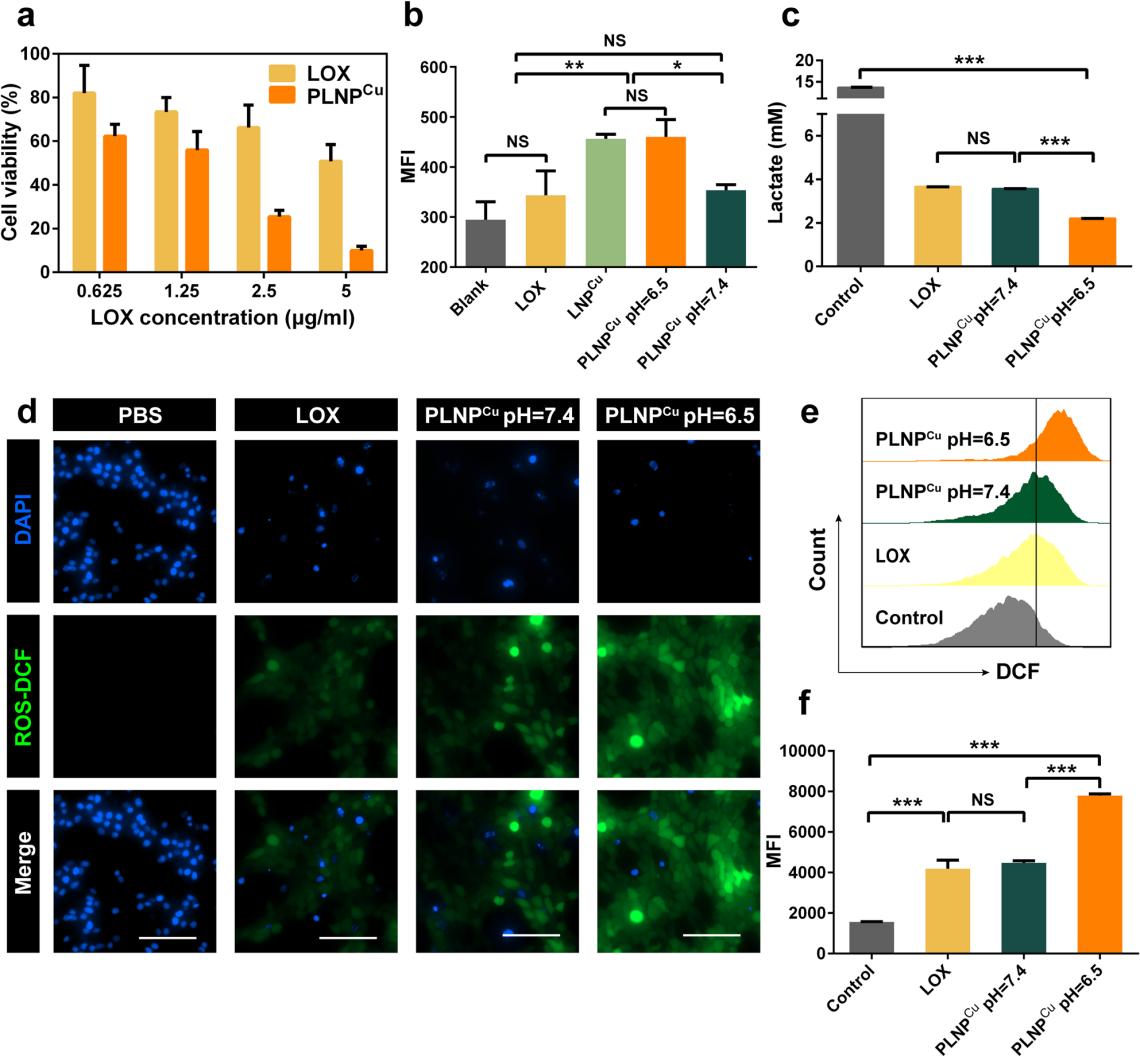
Intracellular behaviors of PLNP^Cu^. **a** The cytotoxicity of PLNP^Cu^ and LOX against 4T1 cells (n = 5). **b** The flow cytometry analysis of cellular uptake of different agents (LOX@FITC, LNP^Cu^*@*FITC, PLNP^Cu^*@*FITC pH = 7.4 and PLNP^Cu^*@*FITC pH = 6.5) after 3 h of incubation with 4T1 cells. (n = 3) **c** The lactate consumption analysis in 4T1 cell supernatant after treatment with PLNP^Cu^. (n = 3) **d** Fluorescent microscopy image of intracellular ROS generation. Scale bars, 100 μm. **e**, **f** The flow cytometry analysis of ROS generation after treatment with PBS, LOX, PLNP^Cu^ pH = 6.5 and PLNP^Cu^ pH = 7.4. Results were expressed as mean ± SD. The significant difference was calculated via one-way ANOVA analysis (**b**, **c**, **f**). (NS represented not significant, *p < 0.05, **p < 0.01, ***p < 0.001)

As breast tumors exhibit immunosuppression and Warburg phenotype (with strong aerobic glycolysis induced lactate buildup), 4T1 cell line was chosen for further study [24]. We first examined the cytotoxicity of PLNP^Cu^ on 4T1 cells. As shown in Fig. 3a, PLNP^Cu^ at both low and high concentrations induced high cytotoxicity compared with the free LOX group. The higher cell viability upon treatment with PLNP^Cu^ (pH = 7.4) suggested the protective role of the PEG shell at low concentrations. Once the acid-sensitive reaction was triggered, LNP^Cu^ and PLNP^Cu^ (pH = 6.5) showed similar high efficacy for killing 4T1 cells (Additional file 1: Fig. S8). Besides, PBNP^Cu^ exhibited relatively low cytotoxicity even at high concentrations (2.5 µg mL^−1^) (Additional file 1: Fig. S9), which demonstrated that the establishment of an intracellular lactate treatment plant played a critical role in the efficient killing of tumor cells. Then, the cellular uptake of LNP^Cu^ was examined by flow cytometry. 4 h of incubation with LNP^Cu^@FITC appeared to reach the saturation of cellular uptake for comparable fluorescence intensity as that of 3 h, indicating that the optimal incubation time Additional file 1: Fig. S10.  Particularly, both LNP^Cu^ and PLNP^Cu^ (pH = 6.5) had similar high cellular uptakes in 4T1 cells compared to free LOX and PEG-shielded nanoparticles (PLNP^Cu^ pH = 7.4) (Fig. 3b). Taken together, the superior cellular uptake of detached-shell PLNP^Cu^ and establishment of the intracellular lactate treatment plant account for the toxicity of PLNP^Cu^ to 4T1 cells.

Subsequently, the lactate consumption effect of PLNP^Cu^ was evaluated at the cellular level. Notably, the PLNP^Cu^ (pH = 6.5) group showed the lowest levels of lactate among the experimental groups (Fig. 3c), suggesting that the lactate adsorption caused by PEI exposure on the surface of PLNP^Cu^ promoted the lactate consumption process, which was consistent with the functions verification results in vitro. It also showed the potent inhibition of intra/extracellular lactate. The ·OH generation induced by PLNP^Cu^ was investigated using 2′,7′-dichlorofluorescein diacetate (DCFH-DA) as a ROS indicator. From the fluorescent microscopy images, 4T1 cells treated with free LOX and PBS showed negligible fluorescence. And the faint fluorescence in PLNP^Cu^ (pH = 7.4) group might be induced by the endocytosis of a small number of PLNP^Cu^ (Fig. 3d). In contrast, stronger green fluorescence was observed in PLNP^Cu^ (pH = 6.5) treated cancer cells (Fig. 3d), indicating the relatively high ·OH levels rather than H_2_O_2_. Since the mean fluorescence intensity (MFI) in PLNP (pH = 6.5) (the detailed information of PLNP were shown in Additional file 1: Fig. S2a) treated 4T1 cells was insufficient as well (Additional file 1: Fig. S11). Consistent with fluorescent microscopy images, flow cytometry results also demonstrated the critical role in triggering ·OH formation of copper ions in PLNP^Cu^ (Fig. 3e, f). Overall, PLNP^Cu^ showed the specific function to convert the lactate into the toxic ·OH product through the intracellular Fenton-like reaction, which indicated the successful construction of the intracellular lactate treatment plant.

### Immunomodulatory effects on macrophages and ICD induction effects of PLNP^Cu^ in vitro

**Fig. 4:**
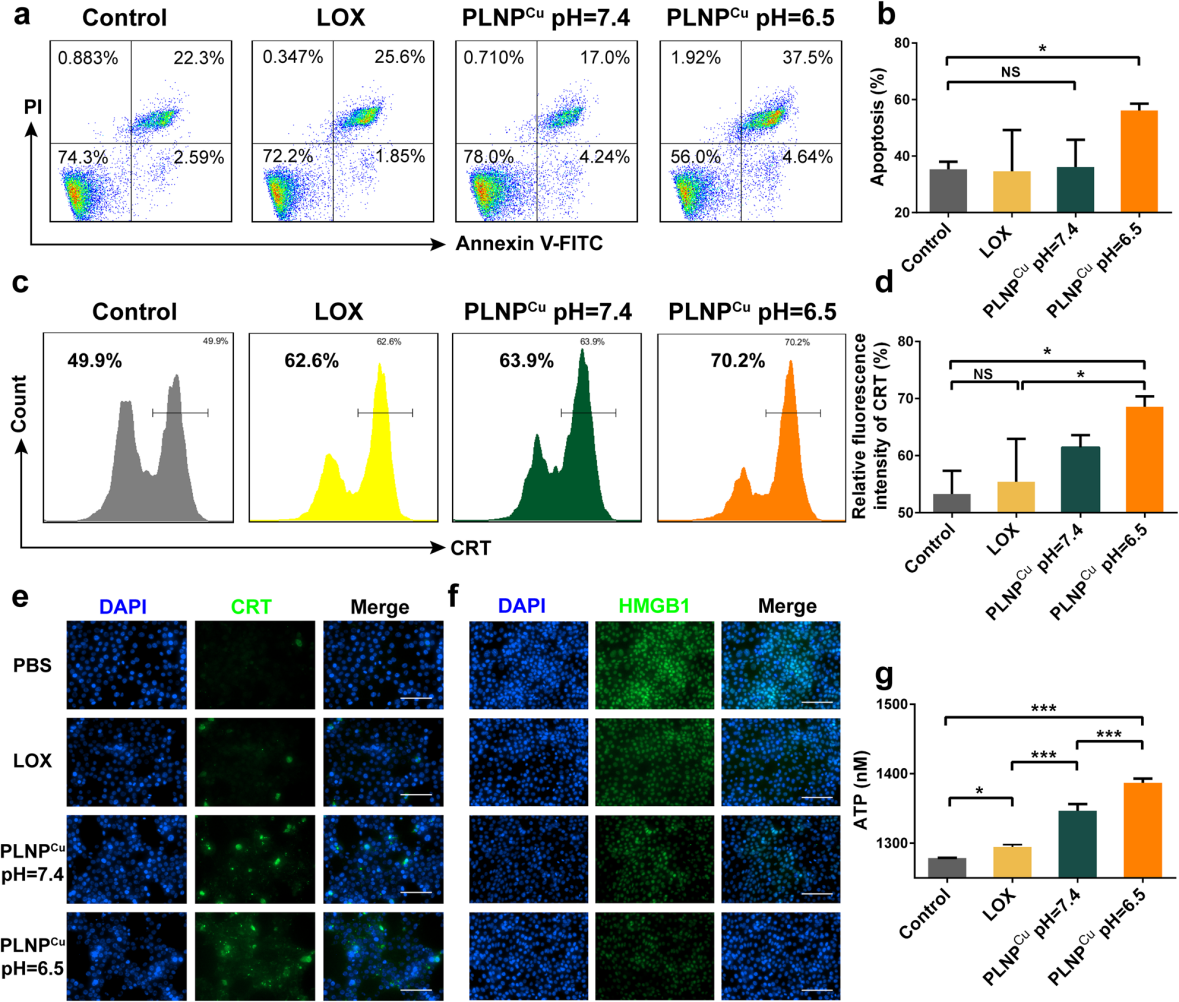
ICD induction effects of PLNP^Cu^ in vitro. **a**, **b** Flow cytometric analysis of apoptosis rate of 4T1 cells after treatment with PBS, LOX, PLNP^Cu^ pH = 7.4 and PLNP^Cu^ pH = 6.5. **c**, **d** The flow cytometric analysis and quantitative results of relative fluorescence intensity of CRT on 4T1 cells. **e** Fluorescent microscopy image of CRT exposure. Scale bars, 100 μm. **f** Fluorescent microscopy image of HMGB1 outflow from the nucleus. Scale bars, 100 μm. **g** The ATP secretion from cancer cells after different treatments. Results were expressed as mean ± SD. The significant difference was calculated via one-way ANOVA analysis (**b**, **d**, **g**). (NS represented not significant, *p < 0.05, **p < 0.01, ***p < 0.001)

·OH, a highly toxic type of ROS, causes oxidative damages to lipids, proteins, and DNA which in turn results in the apoptosis of tumor cells [25]. The apoptosis and necrosis induced by PLNP^Cu^ of 4T1 cells were evaluated by annexin V-FITC and propidium iodide (PI) staining assay. The flow cytometry results showed that the percentage of late apoptosis and necrosis in the PLNP^Cu^ (pH = 6.5) group (56.15%) was nearly 2-fold than other groups (Control ~35.32%, LOX ~34.63%, PLNP^Cu^ pH = 7.4– 36.16%), demonstrating the build of the lactate treatment plant in tumor cells and the excellent chemodynamic therapy (CDT) performance (Fig. 4a, b).

We further investigated the immunogenic cell death (ICD) level of 4T1 cells induced by the lactate treatment plant. As three representative markers of ICD, the expression of calreticulin (CRT), the efflux of high-mobility group box 1 (HMGB1) from the tumor cell nucleus, and the secretion of adenosine triphosphate (ATP) were evaluated, which were critical for activating the adaptive immune response and anti-tumor T cell immunity [26]. As the results were shown in Fig. 4c and d, the CRT expression on tumor surface after being treated with PLNP^Cu^ (pH = 6.5) increased to 68.57%, which was considerably 1.29-fold higher than that in the control group. However, the positive rate of CRT in the LOX group (~55.40%) was insignificantly increased compared with controls, indicating that intracellular H_2_O_2_ was insufficient to trigger ICD. A similar result was reconfirmed in the immunofluorescence (Fig. 4e). Additionally, HMGB1 is normally localized in the cell nucleus, while the weak green fluorescence at cell nuclei in PLNP^Cu^ (pH = 6.5), demonstrating the large quantities of HMGB1 extracellular released compared with the PBS treatment cells (Fig. 4f). For ATP secretion, PLNP^Cu^ treatment resulted in higher ATP in the cellular supernatant (Fig. 4g). Notably, it was Fenton-like chemistry driven by cuprous ions that were the main reason for ICD, based on the results of the lower ATP release in PLNP (pH = 6.5) group compared with PLNP^Cu^ (pH = 6.5) (Additional file 1: Fig. S12). The evidence above suggested that the intracellular lactate treatment plant activated by PLNP^Cu^ converted lactate into anti-tumor ·OH, thereby producing considerable effects on ICD induction in tumor cells, paving the way for anti-tumor immune responses.

**Fig. 5:**
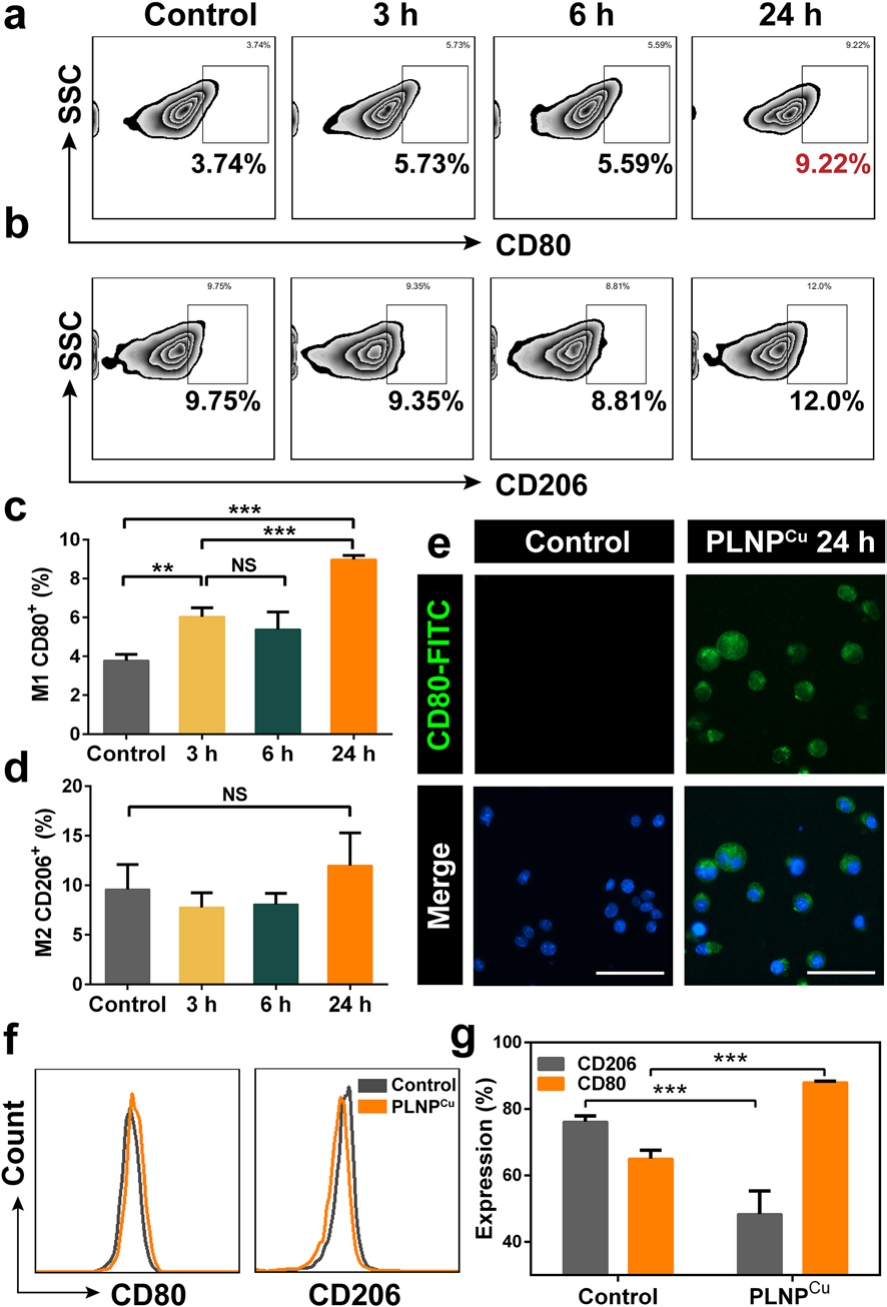
Immunomodulatory effects on macrophages of PLNP^Cu^ in vitro. **a**,** c** The flow cytometric analysis and quantitative results of CD80 (M1 macrophages marker) on RAW 264.7 macrophages after incubation with PLNP^Cu^ for different times (3, 6, 24 h). **b**,** d** The flow cytometric analysis and quantitative results of CD206 (M2 macrophages marker) on RAW 264.7 macrophages after treatment. **e** Immunofluorescence examination of RAW 264.7 macrophages after incubation with PLNP^Cu^ for 24 h. Scale bars, 50 μm. **f**, ** g**The flow cytometric analysis and quantitative results of CD80 and CD206 on M2-macrophages after treatment. Results were expressed as mean ± SD. The significant difference was calculated via one-way ANOVA analysis (**c**, **d**, **g**). (NS represented not significant, *p < 0.05, **p < 0.01, ***p < 0.001)

As one of the components of innate immunity in TME, macrophages are the major immune component of leukocyte infiltration in the tumor [27]. Unfortunately, lactate efflux associated with lactate accumulation and acid microenvironment suppressed its proliferation and inhibited macrophage M1 polarization [3, 28]. The markers (CD80, CD206) of RAW 264.7 macrophages were analyzed by flow cytometric analysis to determine the polarization after stimulating by the PLNP^Cu^. 1 µg mL^−1^ of LOX was chosen as the safe dose concentration according to the results of the cytotoxicity experiments (Additional file 1: Fig. S13). The results showed that expression of CD80 (M1 macrophages marker) on the cell surface significantly upregulated with the time increase of PLNP^Cu^ stimulation. Especially, it was up to 9.22% after 24 h of stimulation, which was 3-fold higher than the control group (Fig. 5a, c). Again, immunofluorescence images revealed that the PLNP^Cu^ induced significant upregulation of the M1 biomarker CD80. (Green fluorescence signal of FITC labeled CD80, Fig. 5e). However, the RAW 264.7 macrophages displayed an insignificant change in M2-like polarization via analyzing the expression of CD206 (Fig. 5b, d), which may be due to the expression of CD206 both on M0 and M2-macrophage that compromises the alteration of M2 polarization [29]. Therefore, the M0-macrophage was induced by interleukin-4 (IL-4) for generation of the M2 phenotype and used for M2-macrophages in further studies. The results showed that PLNP^Cu^ upregulated expression of CD80 and, notedly, reduced CD206 expression on M2-macrophages (Fig. 5f, g). We also measured the expression of M1 and M2-associated genes via quantitative real-time polymerase chain reaction (qRT-PCR). The results revealed that PLNP^Cu^ exposure significantly induced M1-related markers expression (CD80, TNF-α) while M2-associated markers (CD206, Arg-1) were down-regulated (Additional file 1: Fig. S14). Collectively, these results demonstrated that PLNP^Cu^ induced macrophage M1 polarization in vitro through the consumption of extracellular lactate, which potentiated the antitumoral immune response.

### Distribution and antitumor effects of PLNP^Cu^in vivo

PLNP^Cu^@Cy7 was prepared to investigate the distribution of the PLNP^Cu^ in 4T1-bearing mice. The major organs (i.e., heart, liver, spleen, lung, and kidney) and tumors were collected within 1 h after tail vein injection of agents (Cy7-labeled LOX, PLNP^Cu^@Cy7 respectively) for ex vivo fluorescence imaging analysis. As shown in Additional file 1: Fig. S15, free LOX indicated a non-specific distribution across the normal organs other than the tumor. In contrast, PLNP^Cu^@Cy7 were significantly enriched in tumor tissues, which might be attributed to the enhanced permeability and retention (EPR) effect [30]. And the enrichment of PLNP^Cu^@Cy7 in the liver was significantly lower than the free LOX, which decreased the immune-related adverse effects due to a non-specific enrichment of LOX in the liver. In conclusion, these findings suggested that PLNP^Cu^ improved tumor targeting efficiency and accumulation, thereby decreasing the non-specific tissue distribution.

**Fig. 6:**
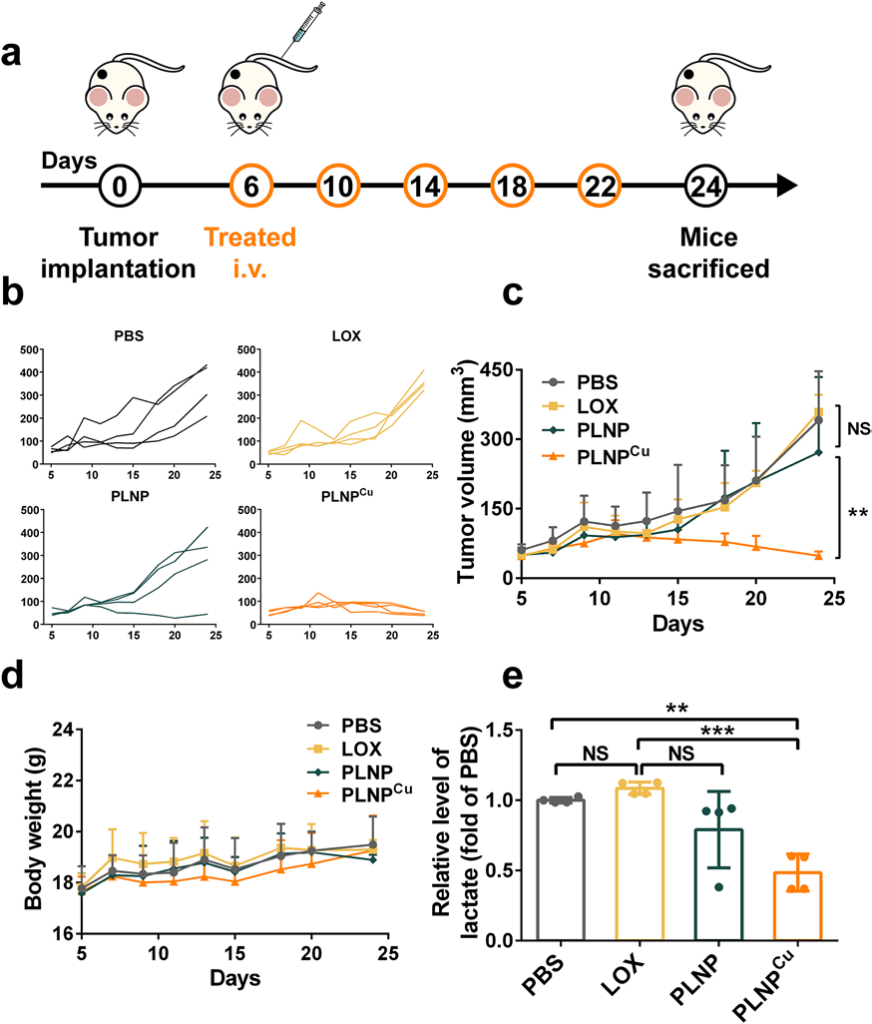
Antitumor effects of PLNP^Cu^ in vivo. **a** The treatment scheme of PLNP^Cu^. **b**,** c** Individual tumor growth curves and average tumor growth curves of the mice with different treatments. **d** Body weight of the mice during the therapy. **e** The lactate consumption effect after treatment in vivo. Results were expressed as mean ± SD. The significant difference was calculated via one-way ANOVA analysis (**c**, **e**). (NS represented not significant, *p < 0.05, **p < 0.01, ***p < 0.001)

The antitumor effect and induction of systemic antitumor immune responses of PLNP^Cu^ were investigated in the 4T1 tumor-bearing mouse model (a weakly immunogenic tumor) (Fig. 6a). After 18 d of administration (five i.v. injections totally), the PLNP^Cu^ group exhibited the most effective tumor inhibition, while tumors showed similarly rapid growth in PBS and LOX groups (Fig. 6b, c). Specifically, PLNP^Cu^ demonstrated 88% tumor inhibition, while the LOX only achieved 17%, and PLNP showed a moderate inhibitory of 37% (Additional file 1: Fig. S16). The tumor volume indicated that PLNP^Cu^ effectively inhibited tumor growth in vivo. The same tendencies of body weight changes (Fig. 6d) and the no obvious tissue damage in H&E staining supported the safety of the PLNP^Cu^ treatment in vivo (Additional file 1: Fig. S17).

The effects of the lactate treatment plant PLNP^Cu^ established in the tumor treatment were further discussed in more detail. Tumor tissues were collected and the intratumoral lactate content was detected after five treatments with PBS, LOX, PLNP, and PLNP^Cu^. The PLNP^Cu^ showed the lowest relative level of lactate (the fold of PBS) in the tumor, at only 0.48, which even reduced to half of the amount for the PBS or LOX group. The result of PLNP (0.79) indicated that thorough consumption and transition of intracellular lactate could counteract the active lactate metabolism in tumors, reflecting in the down-regulation of intra/extracellular lactate levels (Fig. 6e). In general, PLNP^Cu^ showed more effective lactate exhaustion than LOX or PLNP attributed to the effective tumor accumulation of nanoparticles and the well-functioning lactate treatment plant.

**Fig. 7:**
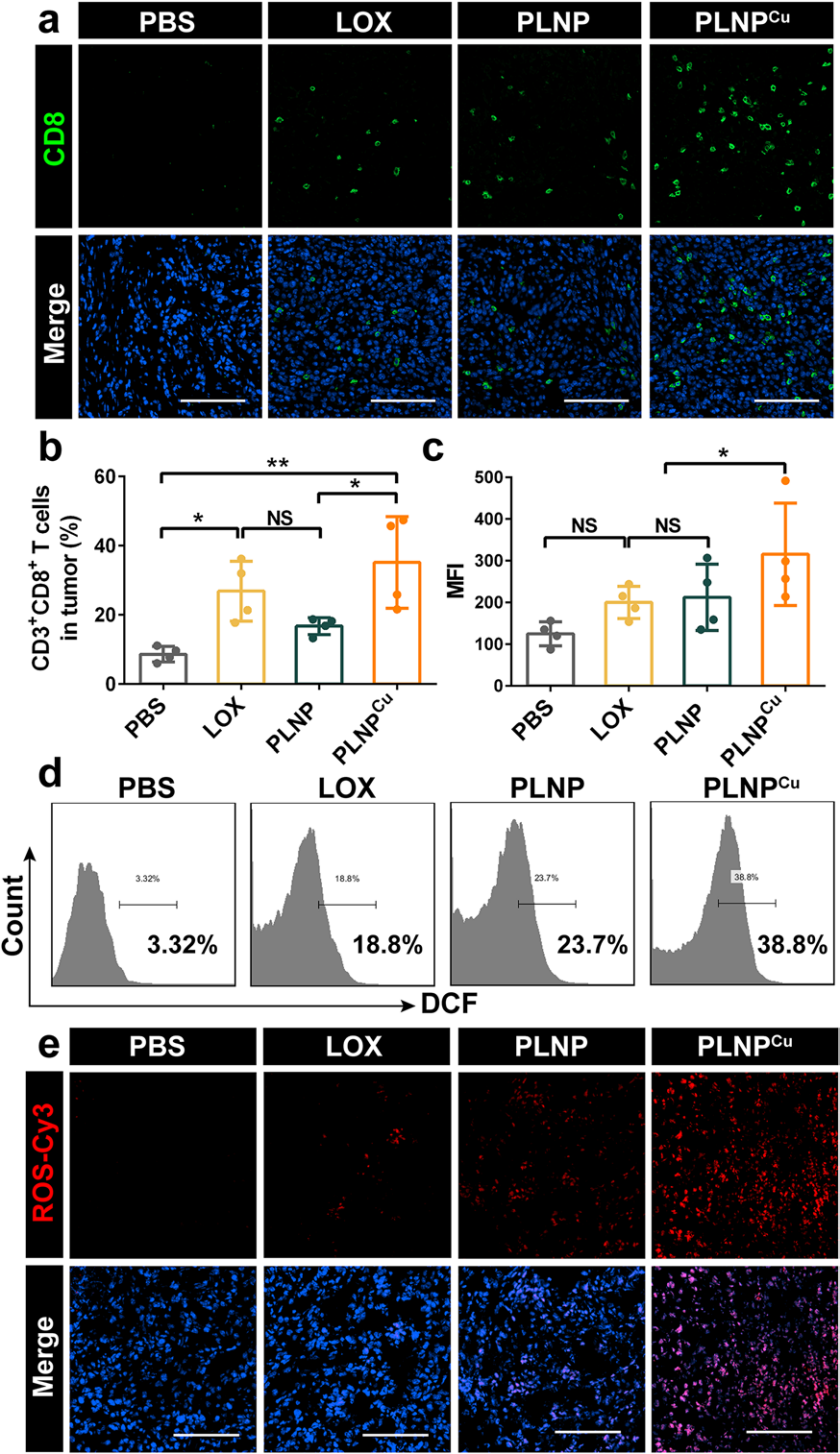
Immunogenic CDT induced an immune-active TME in vivo. **a** Immunofluorescence staining of intratumor infiltrating CD8^+^ T cells after treatments with PBS, LOX, PLNP and PLNP^Cu^. Scale bars, 100 μm. **b** The flow cytometry analysis of tumor infiltration of CD3^+^CD8^+^ T cells. **c**, ** d** The flow cytometry analysis of ROS generation after treatments. **e** Immunofluorescence staining of intratumor ROS generation. Scale bars, 100 μm. Results were expressed as mean ± SD. The significant difference was calculated via one-way ANOVA analysis (**b**, **c**). (NS represented not significant, *p < 0.05, **p < 0.01, ***p < 0.001)

Although these experiments yielded good results, lactate consumption was only the first step of the lactate treatment plant. The generation of ·OH was quantified to validate the plant working properly. The generation of intracellular ROS in the tumor was detected by flow cytometry with the DCFH-DA probe. The MFI of the intracellular ROS in the PLNP^Cu^ group was 3 times higher than that of the PBS. There was no significant difference between the LOX and PLNP groups and, markedly, both were lower than the PLNP^Cu^ group (Fig. 7c, d). The above results confirmed that the PLNP^Cu^ triggered the massive generation of intracellular ROS in 4T1 tumor cells. We estimated that the ROS was therefore mainly toxic ·OH that has a high affinity for DCFH-DA binding rather than H_2_O_2 _[31]. This conclusion was based on the minor amounts of ROS generation in PLNP group, specifically, the third step of the lactate treatment plant, i.e. Fenton-like reaction, was disrupted due to the absence of copper ion. Incidentally, the limited cell uptake and the insufficient toxic ROS generation of free LOX also demonstrated the positive role of the continuous-operated lactate treatment plant in tumor inhibition. The abundant bright red fluorescence was observed in confocal laser scanning microscope (CLSM) images of frozen tumor tissue sections, consistent with the flow cytometry results respectively (Fig. 7e). Together, the lactate treatment plant establishment triggered by PLNP^Cu^ exhibited the most effective lactate exhaustion and tumor inhibition.

### Anti-tumor immune activation in vivo

Encouraged by the well-functioning lactate treatment plant in tumor, we subsequently monitored its anti-tumor immune responses. For this purpose, the typical cell types of cellular immunity (T cells) and innate immunity (macrophages, Tregs) were detected. The percentage of cytotoxic T lymphocytes (CTL, CD3^+^CD8^+^ T cells) infiltration in tumors after PLNP^Cu^ treatment was significantly increased compared with the PBS (4.06-fold higher) and PLNP (2.09-fold higher). There was a non-significant CTL proportion difference between LOX and PLNP groups. Although the slight upregulation of CTL was found in LOX (36.2%), PLNP^Cu^ showed the highest CTL proportion (1.30 times of LOX group) (Fig. 7b). The same results were observed in CLSM images, in which the copious green fluorescent signal at 488 nm confirmed higher CD8^+^ T cell infiltration in PLNP^Cu^ group (Fig. 7a). Furthermore, a typical cytokine (interferon-γ, IFN-γ) of immune activation in tumor tissues was detected. As expected, tumor tissues of PLNP^Cu^ group presented the highest level of IFN-γ after treatment (Additional file 1: Fig. S18). As it is well known, lactate production by cancer cells affects M2-like TAM polarization, which was associated with tumor growth, metastasis, and immunosuppression [3, 32]. According to tumor infiltration macrophages analysis, there was a significant increase in M1-like macrophages after PLNP^Cu^ treatment (Additional file 1: Fig. S19). In addition, regulatory T cells (Tregs) in TME inhibit effector T cells and the activation and proliferation of CD8^+^ T cell by consuming IL-2 and releasing perforin and granzyme [33, 34]. PLNP^Cu^ significantly lowered the number of tumor-infiltrating Tregs in immunofluorescence staining results (Additional file 1: Fig. S20). All the above results suggested that immune response activation at the tumor site was triggered through the establishment of the “lactate treatment plant” by PLNP^Cu^.

**Fig. 8:**
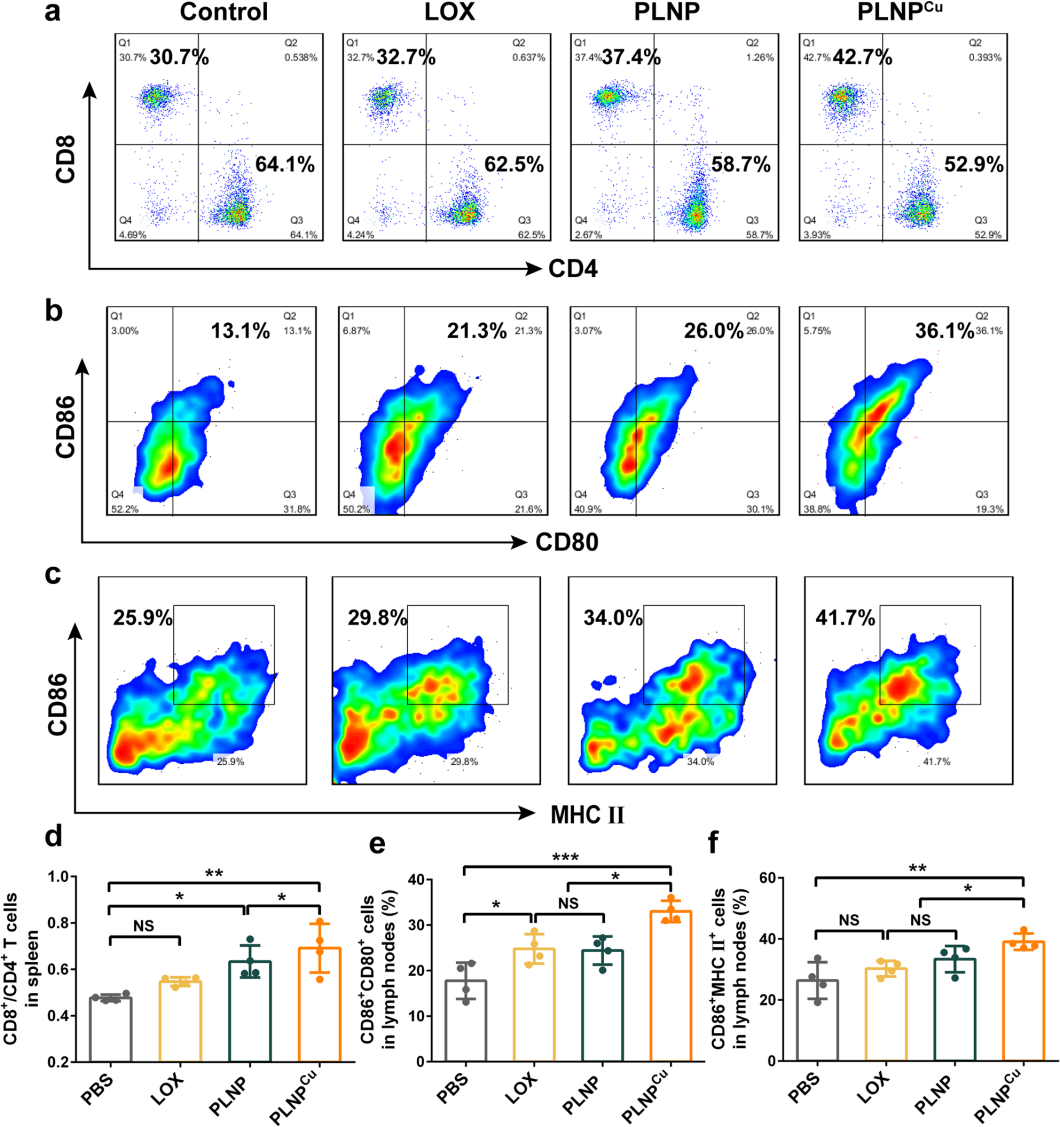
Immune activation by PLNP^Cu^ in vivo. **a** The flow cytometric images of the CD4^+^ T cells and CD8^+^ T cells in spleen (gated on CD3^+^ T cells). **b** The flow cytometric images of the CD80^+^CD86^+^ DCs in LNs (gated on CD11^+^ DCs). **c** The flow cytometric images of the CD86^+^MHC II ^+^ DCs in LNs (gated on CD11^+^ DCs). **d** The flow cytometric quantification of the rate of CD8^+^/CD4^+^ T cells in spleen. **e** The flow cytometric quantification of the CD86^+^CD80^+^ DCs in LNs. **f** The CD86^+^MHC II^+^ DCs in LNs. Results were expressed as mean ± SD. The significant difference was calculated via one-way ANOVA analysis (**d**–**f**). (NS represented not significant, *p < 0.05, **p < 0.01, ***p < 0.001).

The systemic anti-tumor immune response induced by PLNP^Cu^ was also explored. The spleen, containing abundant immune cells, is the largest immune organ and the immune center of the body in supporting anti-tumor immune response activation [35]. We analyzed the phenotype and quantity of spleen T lymphocytes. As shown in the flow cytometry results, CD8^+^ T cells significantly increased in PLNP^Cu^ treatment group, the proportion of CD4^+^ T cells was downregulated (Fig. 8a). Remarkably, PLNP^Cu^ increased the rate of CD8^+^/CD4^+^ T cells from 0.47 of PBS to 0.69 (Fig. 8d). Dendritic cells (DCs) present the tumor antigen to T cells, playing critical roles in pathogen sensing and initiation of anti-tumor immune responses. The DC maturation in draining lymph nodes (DLNs) including tumor DLNs was analyzed via flow cytometry. By qualitative and quantitative assays, the expressions of the DC maturation marker MHC II, CD86, and CD80 were up-regulated, confirming PLNP^Cu^ promotes DC maturation in DLNs (Fig. 8b, c, e and f). Together, all the immune analysis results suggesting that PLNP^Cu^ activated the immune system for efficient inhibition of tumor cell growth.

## Conclusions

In summary, a pH-responsive nanosystem PLNP^Cu^ was constructed with synergistic anti-tumor effects via the combinatorial treatment with both metabolic therapy and immunotherapy. PLNP^Cu^ delicately used the tumor cell phsiology and TME to build the “lactate treatment plant”, where the raw materials (H_2_O_2_ that the enzymatic products of LOX, cuprous ions from nanosystem) processed into toxic ·OH products through Fenton-like reactions for ICD, and then the immune system was activated for tumor inhibition. Meanwhile, the intra/extracellular lactate exhaustion remodeled the immunosuppressive TME, inducing TAM polarization to M1 phenotype and arousing the activity of immune cells around the tumor cells. Importantly, the 88% tumor inhibition was achieved with the PLNP^Cu^ treatment alone without the introduction of additional agents.

## Supplementary Information

**Additional file 1. **Supporting information including materials, methods, additional figures and tables.

## Data Availability

All data generated or analyzed during this study are included in this published article and its Additional file 1.
