# Supplementary material for: Nanofactory for metabolic and chemodynamic therapy: pro-tumor lactate trapping and anti-tumor ROS transition
Source: J Nanobiotechnology. 2021 Dec 18;19:426. doi: 10.1186/s12951-021-01169-9 (PMC8684183; doi:10.1186/s12951-021-01169-9)
Supplement: Supplementary file 1 — Additional file 1. Supporting information including materials, methods, additional figures and tables. [file 12951_2021_1169_MOESM1_ESM.docx]

Additional file 1

Nanofactory for metabolic and chemodynamic therapy: pro^-^tumor lactate trapping and anti-tumor ROS transition

Ruiqing He^1#^, Jie Zang^1#^, Yuge Zhao^1^, Ying Liu^1^, Shuangrong Ruan^1^, Xiao Zheng^1^, Gaowei Chong^1^, Dailin Xu^1^, Yan Yang^1^, Yushan Yang^1^, Tingting Zhang^1^, Jingjing Gu^1^, Haiqing Dong^2^*, and Yongyong Li^1^*

^1^ Shanghai Skin Disease Hospital, The Institute for Biomedical Engineering & Nano Science, School of Medicine, Tongji University, Shanghai 200092, China.

^2^ Shanghai East hospital, School of Medicine, Tongji University, Shanghai 200092, China

Correspondence: [yongyong_li@tongji.edu.cn](mailto:yongyong_li@tongji.edu.cn); inano_donghq@tongji.edu.cn

^#^ R. He and J. Zang contributed equally to this work

| co-authors | E-mail |
| --- | --- |
| Ruiqing He | 756810884@qq.com |
| Jie Zang | 2457232961@qq.com |
| Yuge Zhao | 1528799301@qq.com |
| Ying Liu | ying_liu97@163.com |
| Shuangrong Ruan | 17369278712@163.com |
| Xiao Zheng | 13395830229@163.com |
| Gaowei Chong | 1931235@tongji.edu.cn |
| Dailin Xu | xudailinxdl@126.com |
| Yan Yang | 1350559526@qq.com |
| Yushan Yang | 916359853@qq.com |
| Tingting Zhang | 1778233513@qq.com |
| Jingjing Gu | 15837802819@163.com |
| Haiqing Dong | inano_donghq@tongji.edu.cn |
| Yongyong Li | yongyong_li@tongji.edu.cn |

The PDF file includes:

Materials and Methods

Table S1. List of antibodies

Table S2. List of primer sequences

Figure S1-S20

Experimental Section

**Materials:**

Branched Polyethyleneimine (PEI) with molecular weight of 25,000 Da (PEI_25K_), collagenase and Bovine serum albumin (BSA) were purchased from Sigma-Aldrich (Shanghai). Lactate Oxidase from *Pediococcus sp.* (LOX) were purchased from ShanghaiyuanyeBio-Technology Co.Ltd.. CuCl_2_·2H_2_O (AR), L-Lactic Acid (≥90%), Sodium hydroxide(≥98%), Hydrogen peroxide solution(AR, 30 wt. %), Methylene blue (MB), Glutathione (Reduced) (GSH) (98%) and dimethyl sulfoxide (DMSO) were supplied from Aladdin Chemistry Co. Ltd.. DF-PEG-DF with molecular weight of 2,000 Da (PEG_2K_) were purchased from Tanshtech (Guangzhou). Lactic Acid Assay Kit was provided by Nanjing Jiancheng Bioengineering Institute (China). Fetal bovine serum (FBS), Dulbecco’s modified Eagle’s medium (DMEM), penicillin-streptomycin and trypsin were supplied by Gibco Invitrogen. 2′,7′-dichlorofluorescein diacetate (DCFH-DA) were obtained from Shanghai feiduo Biotechnology Co., Ltd.. Paraformaldehyde (4%) was obtained from DingGuo Chang Sheng Biotech. Dialysis membranes were purchased from Tian Nan Technology Co., Ltd.. DAPI Staining Solution, Hydrogen Peroxide Assay Kit, Bradford Protein Assay Kit, Annexin V-FITC Apoptosis Detection Kit, ATP detection kit and Cell Counting Kit-8 were purchased from Beyotime Institute of Biotechnolog. Recombinant Mouse IFN-γ was purchased from Novoprotein Co., Ltd.. Zombie Violet Fixable Viability Kit, Intracellular Staining Perm Wash Buffer (10×) and Fixation Buffer were purchased from Biolegend. ROS reactive oxygen Assay Kit was supplied from Servicebio. Trizol kit, Hifair^®^ Ⅲ 1st Strand cDNA Synthesis SuperMix Kit, Hieff^®^ qPCR SYBR Green Master Mix were purchased from Yeasen Biotechnology (Shanghai) Co., Ltd..

**Synthesis and characterization of PEI-PEG**

The PEI-PEG was synthesized and the characterization of its responsive capability was according to the previous literature.[1] PEI (1 mg·mL^−1^) and OHC-PEG-CHO (1 mg·mL^−1^) were dissolved in H_2_O and reacted for 30 min at pH 7.8. Then adjusted pH of the solution by HCl to 7.4 and 6.5, respectively, and reacted for 10 min. After freeze-drying, the product was redissolved with D_2_O. Then the ^1^H NMR spectra through a Bruker AVANCE III HD 600 MHz NMR spectrometer were detected.

**Preparation and characterization of nanoparticles**

Three kinds of nanoparticles (LNP, LNP^Cu^, BNP^Cu^) were prepared through a facile procedure. Briefly, the molar ratio of PEI (1 mg·mL^−1^), Cu^2+^ or not (0.01 mg·mL^−1^) and LOX/BSA (10 mg·mL^−1^) was 10:2:1. The CuCl_2_ solution and LOX/BSA solution were dropwise added in turn to PEI solution under stirring for 10 min to obtain the original nanoparticles. Afterward, pH-responsive nanoparticles (PLNP, PLNP^Cu^, PBNP^Cu^) were obtained by adding OHC-PEG-CHO (4 mg·mL^−1^) to pre-prepared original nanoparticles solution with a mass ratio of 2:1 for OHC-PEG-CHO/PEI at pH 7.8. All the above reactions were carried out in DD H_2_O. After 30 min slow stirring at room temperature, the pH-responsive nanoparticles were collected by centrifugation and washed with ultrapure water (5500 rpm, 10 min). The lower fluid was collected to estimate LOX loading by Bradford Protein Assay Kit.

The zeta potential and particle size of nanoparticles at different pH values (pH 7.4, 6.5 and 5.5, respectively) were measured by dynamic light scattering (DLS, ZS90, Malvern) at room temperature. The morphological characteristic of the nanoparticles was observed by transmission electron microscope (TEM).

**Responsive capability of** **PLNP^Cu^**

The zeta potential and particle size alteration were reflexes of the responsive capability of PLNP^Cu^. Briefly, PLNP^Cu^ was incubated in DD H_2_O at different pH values (pH 7.4, 6.5, 5.5, respectively) at 37°C. The samples were collected for measuring by DLS at the given time points. In order to explore the acidity-triggered release of LOX. The LOX was collected using the ultrafiltration tube [Pall Corporation, molecular weight cut-off (MWCO), 300 kDa] at set time points (0 h, 1 h, 2 h, 4 h, 8 h, 20 h), and examined by Bradford Protein Assay Kit.

**Hemolysis evaluation of PLNP^Cu^**

The 5% red blood cell (RBC) suspension was prepared first. A series concentration of PEI (1.25 to 20 μg·mL^−1^) PLNP^Cu^ solutions dispersed in PBS were mixed with the equal volume 5% RBC suspension. Equivalent PBS and water were used as the negative and positive controls, respectively. After keeping at 37℃ for 3 h, the absorbance of supernatants collected by centrifugation (3000 rpm, 1 min) at 540 nm was measured by UV-visible spectroscopy (Varian, Ltd., Hong Kong). The hemolysis ratio was calculated according to the following formula.

$$\text{Hemolysis ratio(\%) =}\frac{\text{A}^{\text{sample}}\text{ - A}^{\text{negative}}}{\text{A}^{\text{positive}}\text{ - A}^{\text{negative}}}\text{ × 100\%}$$

**Lactate adsorption**

The lactate adsorption rate was determined by the lactate concentration mixed with PEI. Briefly, the PEI, BNP^Cu^, PBNP^Cu^ pH 7.4, PBNP^Cu^ pH 6.5 respectively, were dispersed in DD H_2_O at an identical amount of PEI (250 μg). DD H_2_O pH 7.4 was acted as the negative control. Then, the above solution was mixed with NaL (0.4 mmol·mL^−1^, 15 μL) respectively, and shaken overnight at 37℃. Next, the mixed solution was placed in the dialysis bag (5 kDa) and fully dialyzed for 4 h, and the dialysate of different groups was collected for lactate analysis by Lactic Acid assay Kit. The lactate adsorption rate was calculated by the formula: the lactate adsorption rate = molar of lactate after dialysis/before.

**Lactate degradation**

The lactate degradation effect of PLNP^Cu^ was studied under incomplete or sufficient reaction condition. At first, the lactate exhaustion of LNP^Cu^ or BNP^Cu^ at different time points was analyzed. The LNP^Cu^ or BNP^Cu^ (LOX or BSA = 20 μL, 10 mg·mL^−1^, pH 7.4) was incubated in DD H_2_O at 37°C with NaL (0.4 mmol·mL^−1^, 20 μL) solution added and then collected the supernatant at preset time intervals (0, 15, 30, 45, 60, 90, 120 min) for lactate qualification by Lactic Acid Assay Kit. For incomplete exhaustion, the LNP^Cu^ at different pH values (pH 7.4, 6.5, 5.5, respectively) or LOX pH 7.4 (12.8 μg) was incubated in DD H_2_O at 37°C. Then added NaL (0.4 mmol·mL^−1^, 12.5 μL) solution and collected the supernatant as the initial concentration. After being incubated for 10 or 20 min, the supernatant was collected and immediately detected by Lactic Acid Assay Kit. For sufficient exhaustion, the LNP^Cu^ or LOX (12.8 μg) at different pH values (pH 7.4, 6.5, 5.5, respectively) was incubated in DD H_2_O at 37°C. After being incubated with NaL (0.4 mmol·mL^−1^, 20 μL) for 2 h, the supernatant was collected and immediately detected by Lactic Acid Assay Kit.

**H_2_O_2_ detection**

The H_2_O_2_ was important production of lactate degradation by LOX. The LNP^Cu^ (LOX = 10 μg) was incubated in DD H_2_O at pH 7.4 and incubated with different concentrations of NaL (0, 5, 10, 20 mM, respectively) for 1 h to affirm the moderate concentration of NaL. And the H_2_O_2_ concentration of the supernatant was detected with the Hydrogen Peroxide Assay Kit. For further validation, the LNP^Cu^ or LOX (12.8 μg) was incubated in DD H_2_O at different pH values (pH 7.4, 6.5, 5.5, respectively) at 37°C. Then added NaL (0.4 mmol·mL^−1^, 12.5 μL) solution and incubated for 2 h. The supernatant was collected and detected by the Hydrogen Peroxide Assay Kit.

**∙OH generation**

The generation of ∙OH was reflected on the ·OH-induced MB indicator degradation caused the change in absorbance. Briefly, the prepared nanoparticles (BNP^Cu^/LNP^Cu^, [Cu] = 0.5 mM) were dispersed in DD H_2_O (pH 7.4) and incubated for 30 min at 37℃. Then added GSH (1 mM or not) into the supernatant for 30 min reaction. Subsequently, the H_2_O_2_ (1 mM) was added with suitable MB indicator solution under the addition of NaL (5 mM) solution. After another 30 min of incubation, the absorbance of the supernatants in different groups at 665 nm was immediately measured by UV-visible spectroscopy.

**Cell culture**

The murine 4T1 breast cancer cells and RAW 264.7 cell lines were cultured in DMEM containing 10% FBS and 1% penicillin-streptomycin. All cells were cultured at 37°C under a humidified atmosphere containing 5% CO_2_.

**Cytotoxicity *in vitro***

4T1 cells and RAW 264.7 cells were seeded in the 96-well plate with a density of 5000 cells per well and cultured for 24 h at 37℃. Then the cells were treated with different concentrations of nanoparticles or LOX for 24 h. Next, the cell viability was analyzed by CCK-8 assay.

**Cellular uptake** ***in vitro***

The cancer cellular uptake behavior of LNP^Cu^*@*FITC at different time points was analyzed by flow cytometry (Guava easyCyte). FITC-labeled LNP^Cu^ were prepared first. In brief, LOX (0.2 mg) dissolved in 75 μL of NaHCO_3_ solution (0.1 M) and mix with 25 μL of FITC solution (4 mg·mL^−1^ in DMSO) in dark. After being shaken at room temperature for 2 h, the FITC-labeled LOX (LOX@FITC) was collected by centrifugation and washed with ultrapure water (3 kDa, 6000 rmp×10 min). LNP^Cu^*@*FITC and PLNP^Cu^*@*FITC were prepared according to the above-mentioned methods. 4T1 cells with a density of 4 × 10^5^ cells per well were seeded in a 6-well plate. After culturing overnight, cells were co-cultured with nanoparticles (LOX 1μg·mL^−1^) for different times (0.5 h, 1 h, 2 h, 3 h, 4 h). The 4T1 cells were collected and quantified by flow cytometry.

**ROS generation *in vitro***

Flow cytometry and fluorescence microscope were used to detect the ROS generation caused by PLNP^Cu^. 4T1 cells were seeded in 6-well plates (5 × 10^5^ cells per well) and cultured for 24 h at 37°C. Then cells were co-cultured with fresh medium containing LOX, PLNP^Cu^ pH 7.4, PLNP pH 6.5, and PLNP^Cu^ pH 6.5 (LOX = 2 μg·mL^−1^) for 3 h. The supernatant was collected for further lactate consumption detection and ATP analysis by ATP assay kit. After washing twice with PBS, cells were stained with DCFH-DA (10 μM) for 20 min and washed 3 times with PBS. The cells were collected by centrifugation (3000 rpm, 1 min) and analyzed via flow cytometry. To visualize ROS generation, after washing off the unbound DCFH-DA, cells were stained with DAPI for 5 min and observed by fluorescence microscope.

**Cell apoptosis *in vitro***

4T1 cells were seeded in 6-well plates (5 × 10^5^ cells per well) and cultured for 24 h. Thereafter, the cells were incubated with LOX, PLNP^Cu^ pH 7.4 and PLNP^Cu^ pH 6.5 (LOX = 2 μg·mL^−1^) for 3 h and were centrifuged at 1000 rpm for 3 min, washed with PBS for 3 times. After resuspending in annexin binding buffer (100 μL), Annexin V conjugated FITC and PI were added to the cell suspension for staining at room temperature for 15 min in the dark and analyzed by flow cytometer (BD Biosciences).

**Immunogenic cell death** ***in vitro***

Flow cytometry and immunofluorescence were used to detect the expression of CRT on 4T1 cells. 4T1 cells were seeded in 6-well plates (5 × 10^5^ cells per well) and cultured for 24 h. Then the supernatant of the cells was displaced by the fresh medium containing different agents (LOX, PLNP^Cu^ pH 7.4 and PLNP^Cu^ pH 6.5) (LOX = 2 μg·mL^-1^) and co-cultured for 2.5 h. After fixing by 1% paraformaldehyde, cells were incubated with the CRT primary antibody for 30 min at 37℃ (Dilute with 5% FBS in PBS as 1:100 volume ratio), and then Alexa488-conjugated monoclonal secondary antibody (1:400) for 30 min. Then the cells were collected by centrifugation (3000 rpm, 1 min) and analyzed via flow cytometry. For visible results, 4T1 cells were seeded on a 24-well plate (2 × 10^5^ cells per well) and cultured for 24 h. The dosage of LOX was 1.5 μg·mL^−1^ and the incubation time was 3 h. After fixing by 1% Paraformaldehyde, cells were incubated with the CRT primary antibody at 4℃ overnight (Dilute with 5% FBS in PBS as 1:100 volume ratio), and then Alexa488-conjugated monoclonal secondary antibody (1:500) at room temperature for 1 h. The cells were stained with DAPI for 5 min and observed by fluorescence microscope.

Intracellular HMGB1 distribution was analyzed by immunofluorescence. 4T1 cells were seeded on a 24-well plate (2 × 10^5^ cells per well) and cultured for 24 h. Thereafter, the cells were incubated with LOX, PLNP^Cu^ pH 7.4 and PLNP^Cu^ pH 6.5 (LOX = 1.5 μg·mL^−1^) for 3 h. After fixing by 1% Paraformaldehyde, 0.5% Triton X-100 was used for permeabilizing for 10 min. Then the cells were incubated with anti-HMGB1 antibody at 4℃ overnight and stained with DAPI for immunofluorescence analysis.

**Macrophages polarization** ***in vitro***

Flow cytometry and fluorescence microscope were used to detect the macrophages polarization induced by PLNP^Cu^. RAW 264.7 cells were seeded in 6-well plates (3 × 10^5^ cells per well) and cultured for 24 h. Then the supernatant of the cells was displaced by the fresh medium containing PLNP^Cu^ pH 6.5 (LOX = 1 μg·mL^−1^) and co-cultured for 3, 6 and 24 h, respectively. Then the RAW 264.7 cells were transferred into EP tubes. After washing twice with PBS, cells were blocked with anti-mouse CD16/CD32 for 10 min and stained using surface marker CD80-FITC antibody at 4℃ for 30 min in the dark. Next, the cells were fixed by fixation buffer for 20 min at room temperature and for subsequently permeabilizing. Afterward, RAW 264.7 cells were incubated with the CD206-PE antibody at 4℃ for 30 min in the dark. Finally, the cells were collected by centrifugation (1900 rpm, 5 min) and analyzed via flow cytometry. For immunofluorescence analysis, RAW 264.7 cells were seeded on a 24-well plate (2 × 10^5^ cells per well) and cultured for 24 h. The dosage of LOX and treatment steps were the same as the above flow cytometry. After the surface marker CD80-FITC staining, the cells were stained with DAPI for 5 min and observed by fluorescence microscope.

**M2-like macrophages polarization *in vitro***

M2-like macrophages (RAW 264.7 cells were cultured in DMEM containing 40 ng·mL^−1^ IL-4) were incubated with or without PLNP^Cu^ (LOX = 1 μg·mL^−1^). At 24 h, the cells were harvested for detection of surface markers as mentioned above and for measurement of gene expression as described below.

Total RNA was isolated from treated macrophages using Trizol kit and reverse transcribed into cDNA with the Hifair^®^ Ⅲ 1st Strand cDNA Synthesis SuperMix Kit according to the manufacturer’s protocol. Then, RT-PCR was performed using Hieff^®^ qPCR SYBR Green Master Mix. The transcriptions were quantified using QuantStudio 7 Flex System. The primer sequences are listed in Table S2.

**Animal model**

BALB/c mice (female, 5-6 weeks) were approved by Shanghai Laboratory Animal Center (SLAC, Shanghai, China) and bred in SPF laboratory at Tongji University. All experiments were carried out in accordance with relevant guidelines of the Institutional Animal Care and Use Committee of Tongji University. The 4T1 tumor-bearing mice were built by subcutaneous injection with 4T1 cells (100 μL, 8 × 10^6^ cells mL^−1^) on the right flank.

**Biodistribution**

To study the tissue distribution of PLNP^Cu^, the mice were intravenously injected with PLNP^Cu^@Cy7 or Cy7-labeled LOX (containing the same amount of LOX, 0.5 mg·kg^−1^) when the volume of the 4T1 tumor grew to 100 mm^3^. The mice were sacrificed 1 h after administration. The tumors and main organs were collected for *ex vitro* imaging.

**Antitumor effect *in vivo***

When the tumor grew to 60 mm^3^, 4T1 tumor-bearing mice were divided into four groups randomly (4 mice per group), PBS, LOX, PLNP, PLNP^Cu^. The mice were treated with different agents at an equivalent dose of LOX (0.4 mg·kg^−1^) through tail intravenous injection every three days (five times in total). The body weight and tumor volume were recorded every two days during the treatment, and the tumor volume was calculated according to the formula : V = width^2^ × length × π/6 ≈ width^2^ × length/2. The tumor inhibition ratio = (final tumor volume of PBS group – final tumor volume of other groups)/ final tumor volume of PBS group. After the treatment, tumors, draining lymph nodes (DLNs) and spleen were collected for further immunological analysis and major organs (heart, liver, spleen, lung, kidney) were dissected for hematoxylin and eosin (H&E) analysis.

**Lactate reduction and immune response of tumor tissue *in vivo***

For lactate evaluation, a moderate volume of tumor tissue (≈50 mg) was fully homogenized and distributed in PBS, and the tumor supernatant was obtained by centrifugation (3000 rpm, 10 min) and detected via Lactic Acid assay Kit. The relative level of lactate = lactate concentration of other group/lactate concentration of PBS group.

For immune response evaluation, the tumor tissues were digested by the preheated DMEM medium (containing 2 mg·mL^−1^ Collagenase IV, 0.1 mg·mL^−1^ DNaseI) and shaken for 1 h (37℃, 150 rpm). Then the tumor suspension was filtered through the nylon cell strainer to obtain the single cells suspension. The cells were centrifuged and washed three times with PBS for further analysis. Before the immune-related staining process, cells were purified via viability dye staining and blocked with the anti-CD16/32 antibody. For intracellular ROS detection, the cells were stained with a DCFH-DA probe for 20 min at 37℃. For infiltration of CD8^+^ T cells, the cells were stained with anti-CD3 and anti-CD8 antibody for 30 min at 4℃. For macrophages analysis, the cells were first stained with anti-CD11b, anti-CD80 and anti- F4/80 antibody for 30 min at 4℃. Then the cells were permeabilized for further anti-CD206 antibody staining. Finally, the cells were washed with PBS and collected for flow cytometry analyses. Simultaneously, the ROS expression and infiltration of CD8^+^ T cells in tumor tissue were also detected by immunofluorescence staining. The IFN-γ concentration in the tumor were detected by ELISA Kits.

**Systemic immune regulation *in vivo***

After lymph nodes being collected and ground, the tissue suspension was filtered through the nylon cell strainer to obtain the single cells suspension. Then the cells were stained with anti-CD11c, anti-CD80, anti-CD86 and MHC-II antibody for DC maturation analysis. Spleen was used to analyze the relative distribution ratio of CD8^+^ and CD4^+^ T cells. Same as the lymph nodes treatment steps, the spleen single cells suspension was obtained after the lysis of red cells. The cells were stained with anti-CD3, anti-CD8 and anti-CD4 antibody. Finally, the cells were washed with PBS and collected for flow cytometry analyses.

**Statistical analysis**

All data in the study were presented as mean ± S.D. The statistical analysis was performed by One-way analysis of variance (ANOVA) with SPSS 20.0 (SPSS Inc., USA). Statistical differences were considered as value of * *p* < 0.05, ** *p* < 0.01, *** *p* < 0.001.

Table S1. Antibodies used for flow cytometry or immunofluorescence in this study

| Antibodies | Company | Catalog No. |
| --- | --- | --- |
| Anti-mouse CD16/CD32 | eBioscience | 14-0161-86 |
| Anti-mouse CD11c (APC) | Biolegend | 117310 |
| Anti-mouse CD86 (PE) | Biolegend | 105008 |
| Anti-mouse CD80 (FITC) | Biolegend | 104706 |
| Anti-mouse CD3 (APC) | Biolegend | 100236 |
| Anti-mouse CD4 (FITC) | Biolegend | 100406 |
| Anti-mouse CD8a (PE Cyanine7) | Biolegend | 100722 |
| Anti-mouse I-A/I-E Antibody (Brilliant Violet 421^TM^) | Biolegend | 107632 |
| Anti-mouse CD45 (PE) | eBioscience | 12-0451-82 |
| Anti-mouse CD206 (PE) | Biolegend | 141706 |
| Anti-mouse/human CD11b (APC) | Biolegend | 101212 |
| Anti-mouse F4/80 (PE/Cyanine7) | Biolegend | 123113 |
| Anti-mouse HMGB1 (Alexa Fluor 488) | Biolegend | 651410 |
| Calreticulin Rabbit Monoclonal Antibody | Beyotime | AF1666 |
| [Alexa Fluor 488-labeled Goat Anti-Rabbit IgG(H+L)](https://www.beyotime.com/product/A0423.htm)  Alexa Fluor 488-labeled Goat Anti-Rabbit | Beyotime  Servicebio | A0423  GB25303 |
| Anti-CD8 | Servicebio | GB13429 |
| Anti-Foxp3 | Servicebio | GB112325 |

Table S2. Primer sequences for real-time q-PCR analysis in this study

| Gene | Forward | Reverse |
| --- | --- | --- |
| GAPDH | CAGGAGAGTGTTTCCTCGTCC | TTCCCATTCTCGGCCTTGAC |
| CD80 | TGCCTTGCCGTTACAACTCT | GTATGTGCCCCGGTCTGAAA |
| TNF-α | CCTCACACTCACAAACCACCA | ATAGCAAATCGGCTGACGGT |
| CD206 | GTGGACGCTCTAAGTGCCAT | GAATCTGACACCCAGCGGAA |
| Arg-1 | AGGGTCCACCCTGACCTATG | TTCCCCAGGGTCTACGTCTC |

**References**

1. Wu JY, Chen J, Feng YJ, Zhang SJ, Lin L, Guo ZP, Sun PJ, Xu CN, Tian HY, Chen XS: **An immune cocktail therapy to realize multiple boosting of the cancer-immunity cycle by combination of drug/gene delivery nanoparticles.** *Science Advances* 2020, **6**.


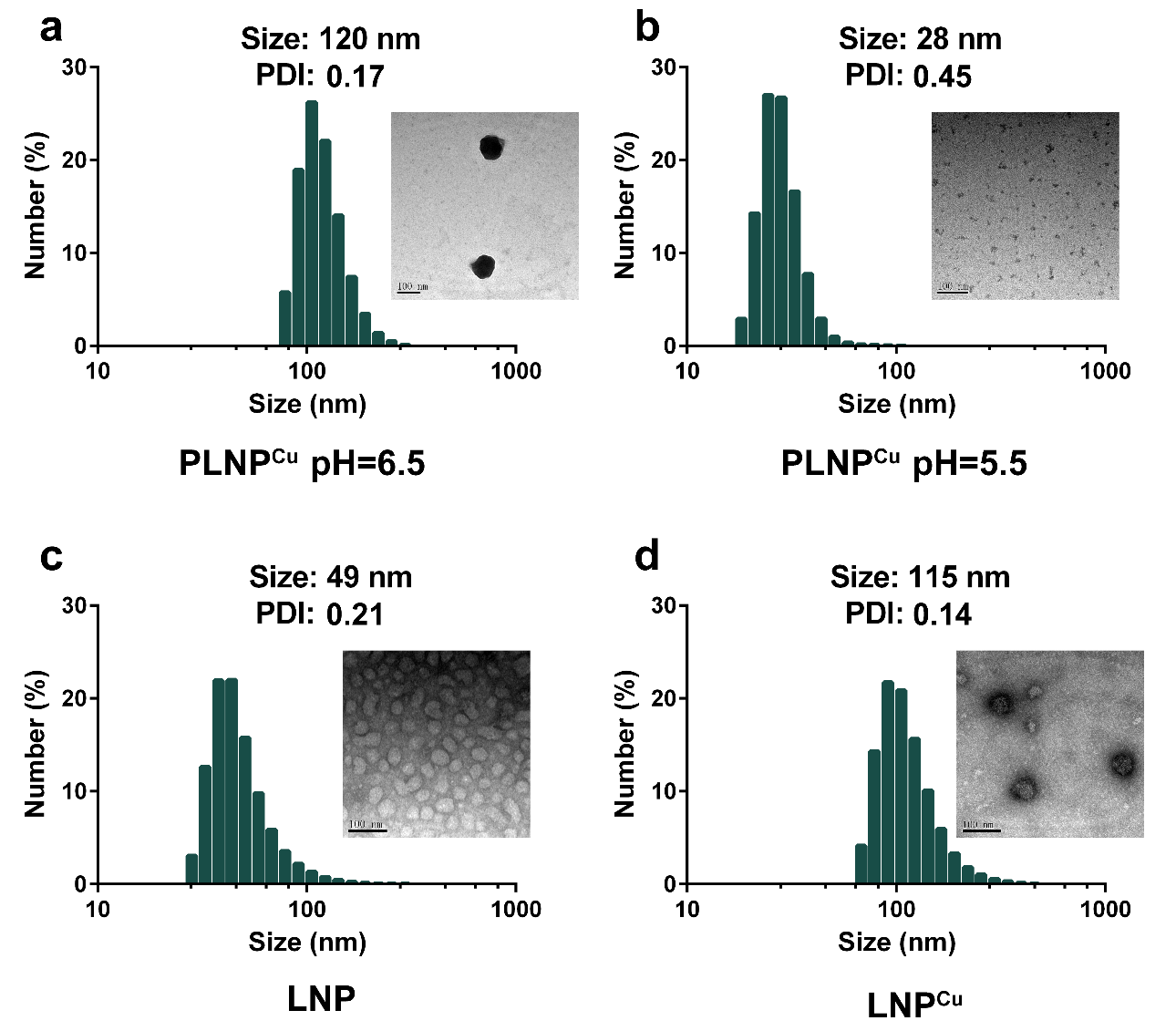


**Fig. S1** TEM images and DLS of **a** PLNP^Cu^ at pH 6.5. **b** PLNP^Cu^ at pH 5.5. **c** LNP at pH 7.4. **d** LNP^Cu^ at pH 7.4.


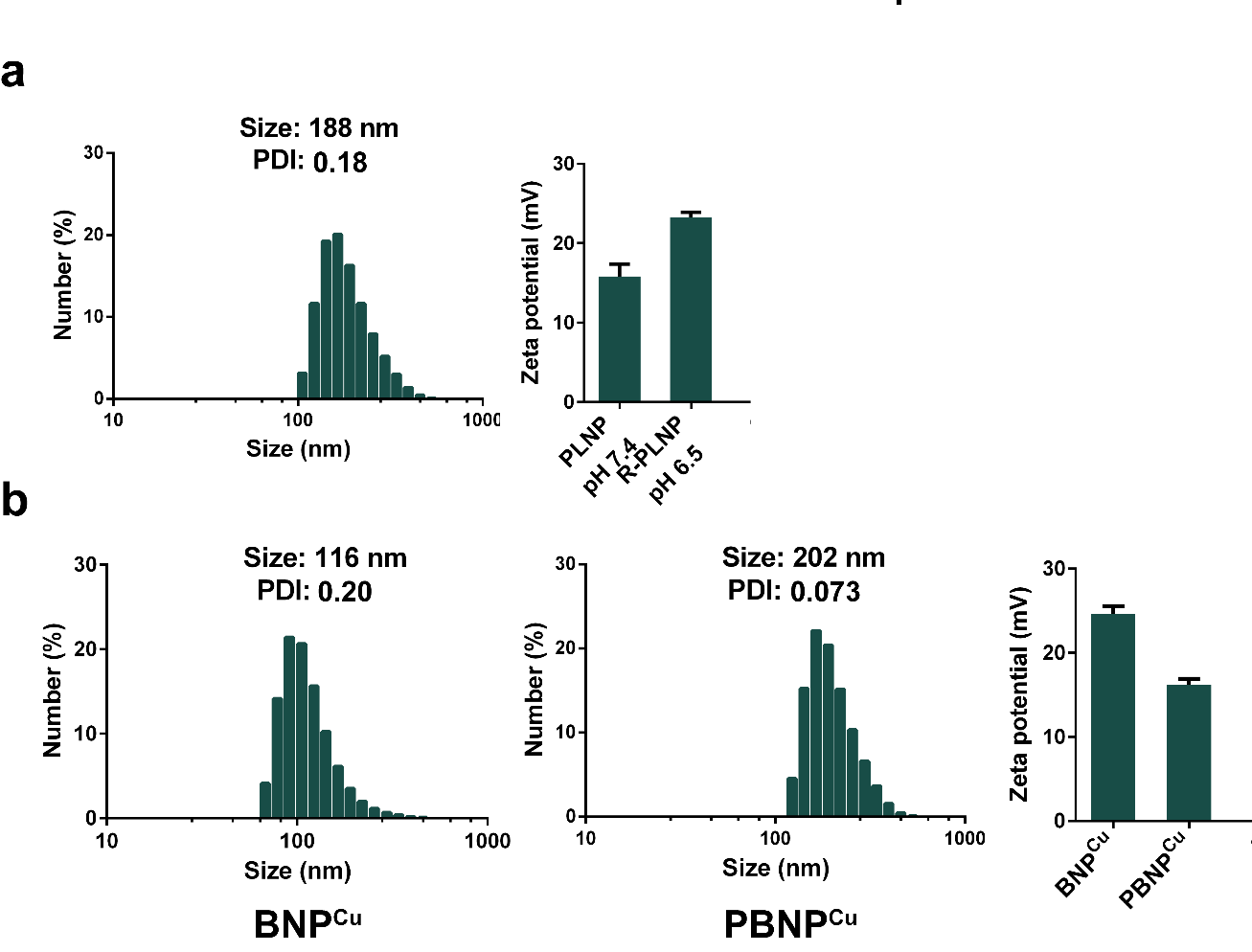


**Fig. S2** **a** DLS and Zeta potential changes of PLNP. **b** DLS and Zeta potential of BNP^Cu^ and PBNP^Cu^ at pH 7.4, respectively.





**Fig. S3** The standard curve of Bradford Protein Assay Kit.


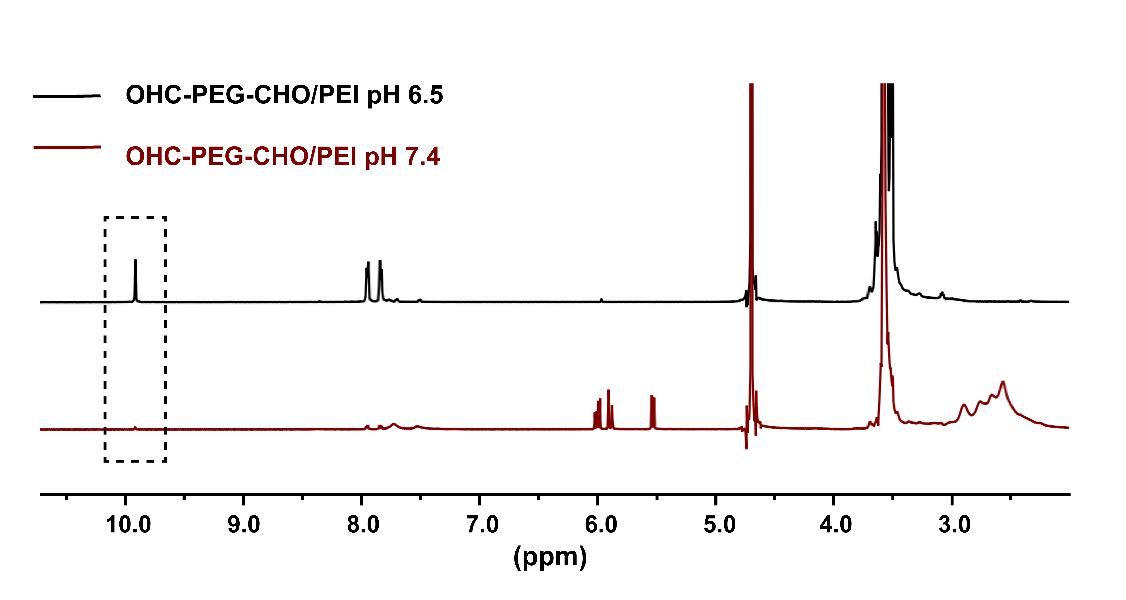


**Fig. S4** The ^1^H NMR spectra of OHC-PEG-CHO/PEI at pH 7.4 and 6.5.





**Fig. S5** The standard curve of Hydrogen Peroxide Assay Kit.





**Fig. S6** The level of H_2_O_2_ generation of LOX at different NaL concentrations.





**Fig. S7** The level of H_2_O_2_ generation of LOX at different pH.





**Fig. S8** The cytotoxicity of LNP^Cu^ pH=6.5, PLNP^Cu^ pH=7.4 and PLNP^Cu^ pH=6.5 against 4T1 cells.





**Fig. S9** The cytotoxicity of PBNP and PBNP^Cu^ against 4T1 cells.


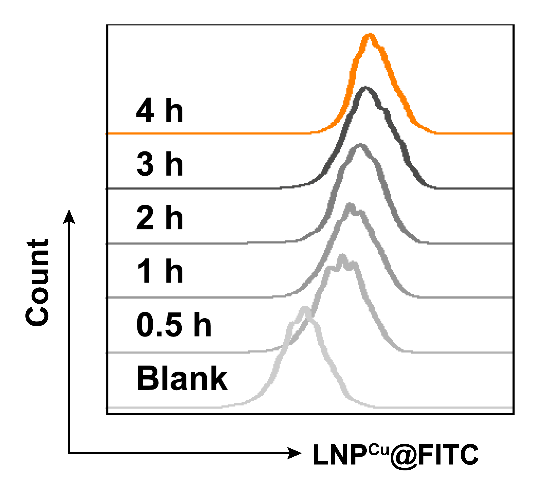


**Fig. S10** The cellular uptake of the LNP^Cu^@FITC after different time of incubation.


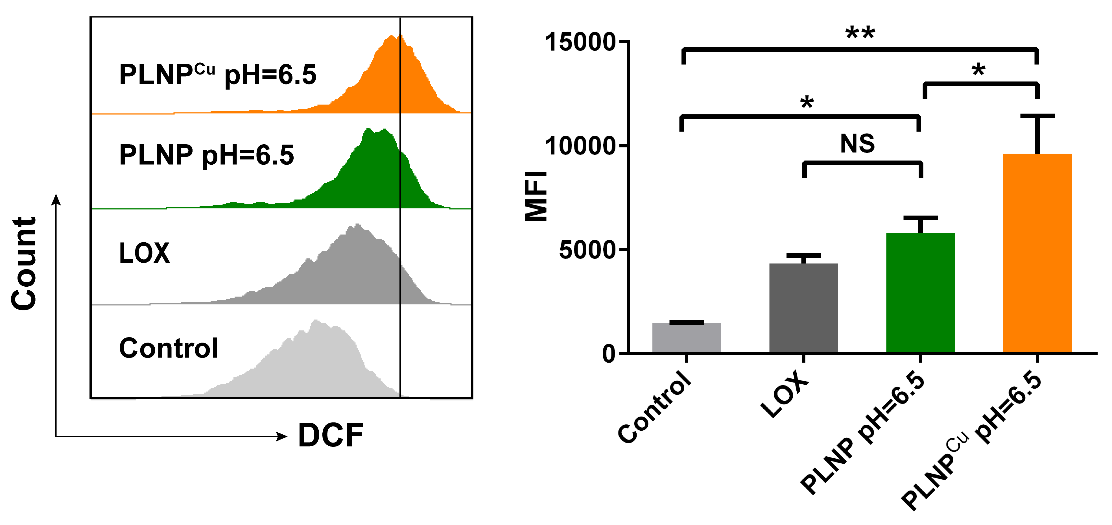


**Fig. S11** Flow cytometry results of the ROS generation.





**Fig. S12** The ATP secretion from cancer cells after different treatment of PLNP pH=6.5 and PLNP^Cu^ pH=6.5, respectively.





**Fig. S13** The cytotoxicity of LOX and PLNP^Cu^ pH=6.5 against RAW 264.7 cells.

^
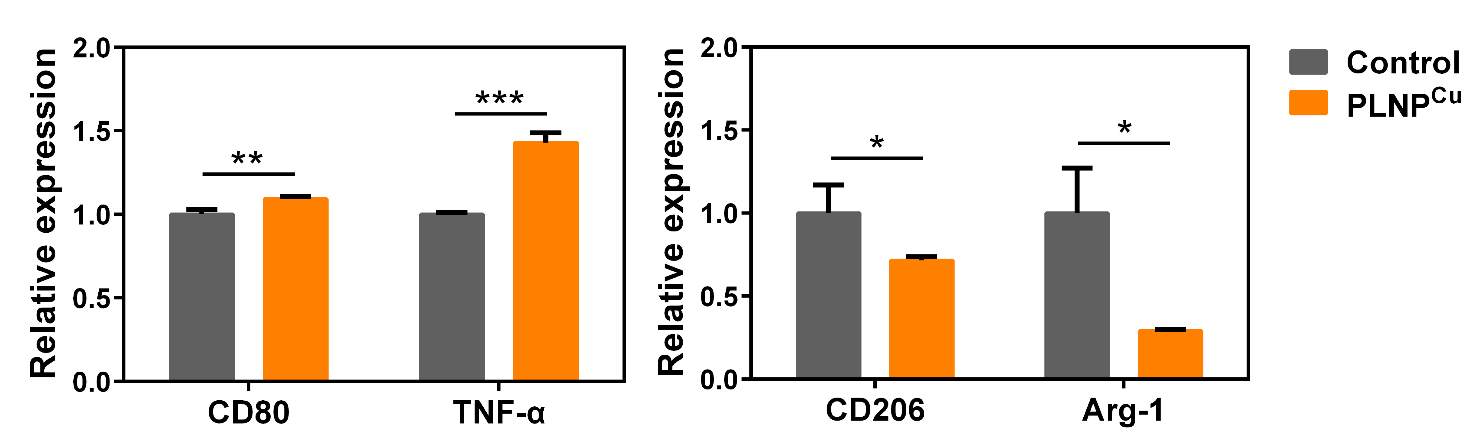
^

**Fig. S14** The qRT-PCR of M1-marker and M2-marker genes expression on M2-macrophages.


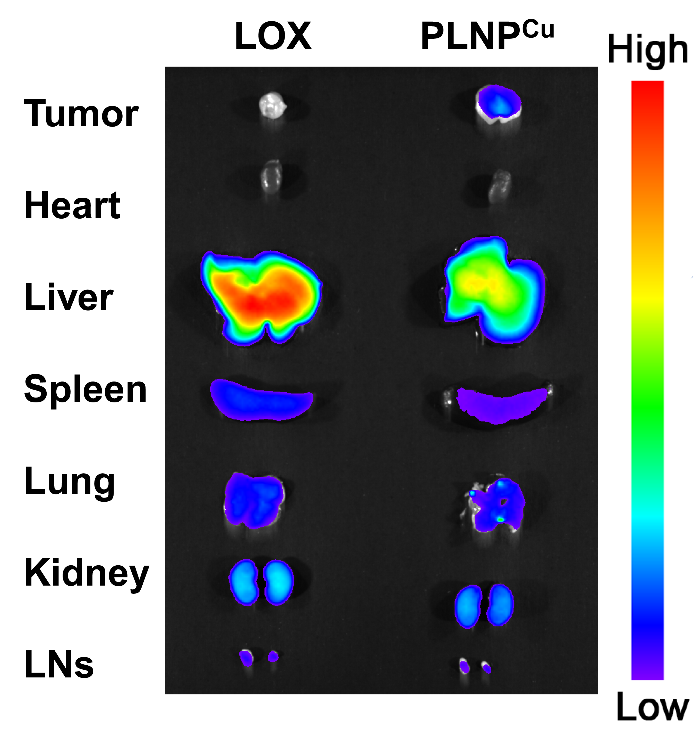


**Fig. S15** The ex vivo fluorescence imaging of major organs and tumors.





**Fig. S16** The tumor inhibition rate of different treatments.


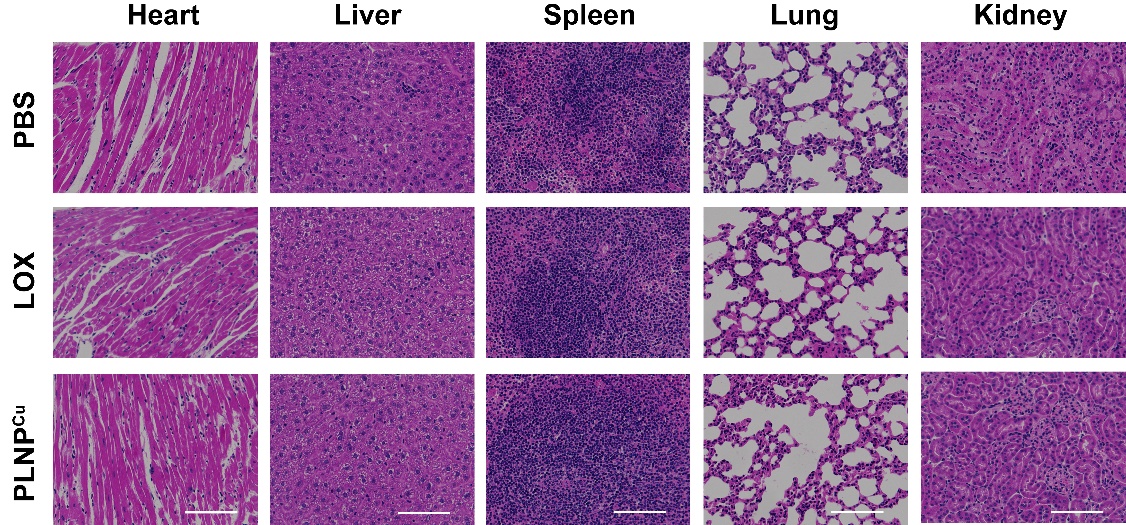


**Fig. S17** H&E staining of main organs after treatment.





**Fig. S18** ELISA analysis for evaluating the IFN-γ content in the tumor site.


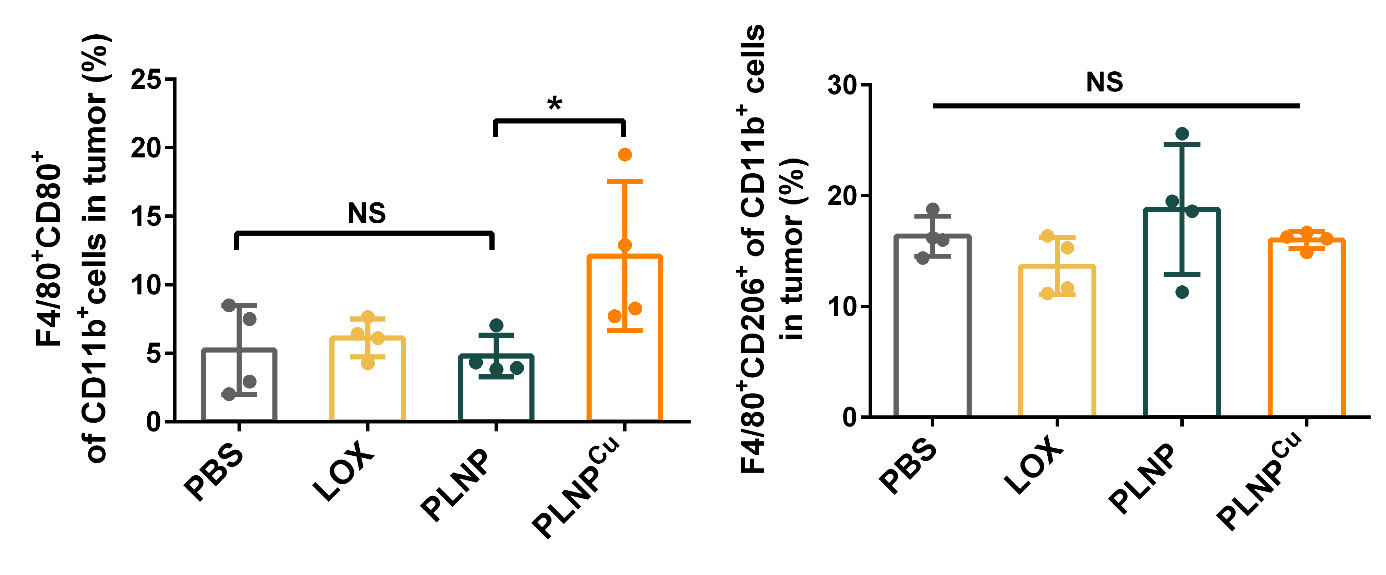


**Fig. S19** Flow cytometry analysis of M1 macrophages and M2 macrophages in the tumor site.


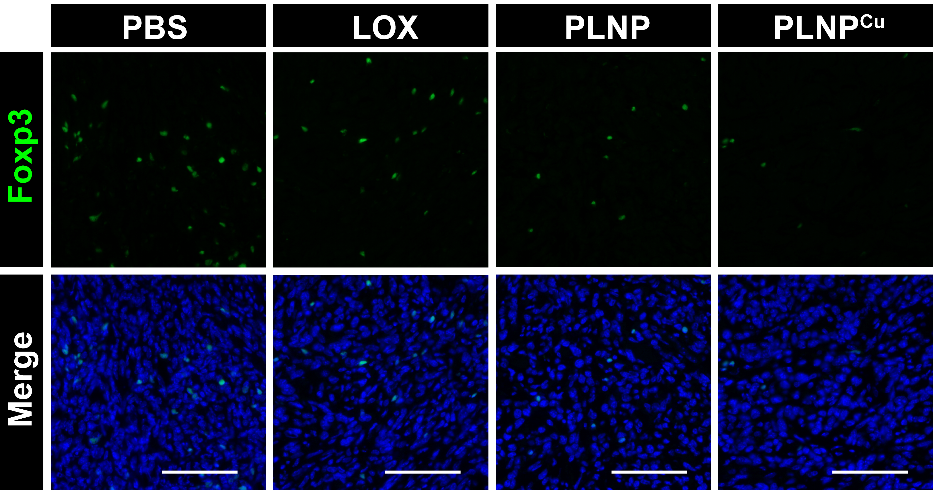


**Fig. S20** Immunofluorescence staining of Tregs in the tumor site. Scale bars, 100 μm.
